# Hierarchical physiological sensitivity of the coral, *Orbicella faveolata*, to suspended sediment and elevated temperature

**DOI:** 10.1007/s10661-026-15969-9

**Published:** 2026-10-13

**Authors:** Keisha D. Bahr, Robert J. Bretzing-Tungate, Briana M. Matthews, Eleanor G. TenBrink, Casey L. Gallagher, Abigail L. Cannon

**Affiliations:** Harte Research Institute, Texas A&M University-Corpus Christi, Corpus Christi, TX 78412 USA

**Keywords:** Suspended sediment, Thermal stress, Coral photophysiology, PAM fluorometry, Respirometry, *Orbicella faveolata*

## Abstract

Coral reefs are increasingly exposed to multiple environmental stressors, including ocean warming and elevated turbidity from coastal development, dredging, and sediment resuspension. Understanding how these stressors affect coral physiology is essential for improving sediment management and protecting reef ecosystems. We investigated the short-term physiological responses of the Caribbean reef-building coral, *Orbicella faveolata*, to suspended sediment and elevated temperature during controlled 72-h laboratory experiments. Coral fragments were first exposed in independent chamber–sump systems to a gradient of target turbidity levels consisting of a control with no added sediment and treatments of 15, 29, and 50 NTU, followed by a factorial experiment evaluating suspended sediment (control or 29 NTU) and temperature (27°C or 30°C). Metabolic oxygen flux was quantified using intermittent-flow respirometry; photophysiological performance was assessed using pulse-amplitude-modulated fluorometry, and tissue and skeletal condition were evaluated using biological and calcification endpoints. Elevated temperature produced the most consistent responses, decreasing maximum photochemical efficiency (Fv/Fm), increasing regulated non-photochemical energy dissipation (YNPQ), and altering respiration. Suspended sediment elicited comparatively limited responses, primarily affecting photophysiological metrics, with modest effects on respiration in the highest-turbidity treatment. No suspended sediment × temperature interaction was detected. Net calcification, symbiont density, chlorophyll concentration, and host protein content remained largely unchanged across treatments, while genotype accounted for significant variation in multiple physiological responses. Collectively, these findings support a hierarchy of short-term physiological sensitivity, with photophysiological metrics showing the most consistent responses, metabolic responses being more limited, and biomass-related and skeletal-growth endpoints remaining largely stable over the 72-h exposure. Integrating photophysiological measurements into monitoring programs may improve early detection of sublethal coral stress during coastal and climate-related disturbances.

**Supplementary Information:**

The online version contains supplementary material available at https://doi.org/10.1007/s10661-026-15969-9.

## Introduction

Coral reefs provide fisheries, coastal protection, tourism, and other ecosystem services but are undergoing rapid ecological change from the combined effects of global climate change and local environmental stressors (Good & Bahr, 2021; Hughes et al., 2018; Spalding et al., 2017; Woodhead et al., 2019). Reef-building corals function as holobionts composed of the cnidarian host, photosynthetic dinoflagellate symbionts, and associated microorganisms that collectively influence metabolism and stress responses (Peixoto et al., 2017; Rädecker et al., 2021). Environmental stress can destabilize this association and cause bleaching, reducing coral energy acquisition and potentially leading to mortality (Douglas, 2003; Weis, 2008).

Thermal stress associated with ocean warming is the primary driver of global coral bleaching events, yet bleaching typically results from interactions among multiple environmental stressors rather than temperature alone (Hughes et al., 2018; Suggett & Smith, 2020; Sully et al., 2019). Recent advances in coral physiology have revealed that environmental stressors can initiate complex cellular responses within the coral holobiont that ultimately lead to symbiont loss (Helgoe et al., 2024). Many bleaching responses involve disruption of symbiont photosynthesis and photochemical processes, which can promote oxidative stress and cellular damage in both symbiont and host tissues (Oakley & Davy, 2018; Warner et al., 1999). Importantly, these early physiological responses often precede visible bleaching or changes in tissue biomass, making photophysiological and metabolic measurements particularly useful for detecting sublethal stress. This mechanistic framework highlights the interconnected nature of coral stress responses and underscores the importance of quantifying early physiological responses to multiple interacting environmental stressors before visible bleaching or tissue loss occurs.

In addition to global stressors such as ocean warming, local declines in water quality can strongly influence coral health and resilience (Good & Bahr, 2021). One of the most widespread local stressors affecting coral reefs is elevated turbidity from suspended particulate matter (SPM), which can arise from terrestrial runoff, coastal development, dredging, and storm-driven sediment resuspension (Bessell-Browne et al., 2017; Erftemeijer et al., 2012; Rogers, 1990). Turbidity is an optical property of water that reflects the scattering and absorption of light by suspended particulate matter and other optically active constituents, thereby reducing the quantity and quality of light available to benthic organisms (Fabricius, 2005; Kirk, 1994). Because light availability directly regulates photosynthesis in Symbiodiniaceae, increased turbidity can alter coral energy acquisition, metabolism, and physiological performance (Anthony & Hoegh-Guldberg, 2003; Grottoli et al., 2018; Rädecker et al., 2021).

It is important to distinguish turbidity from sedimentation because these processes affect corals through different mechanisms. Turbidity describes the optical consequences of suspended material remaining within the water column, whereas sedimentation refers to the process by which particles settle from suspension. Once settled, these particles may accumulate on coral surfaces as sediment, which can physically smother coral tissues, impede gas exchange and heterotrophic feeding, and increase the energetic costs associated with mucus production and sediment rejection (Rogers, 1990; Tuttle & Donahue, 2022; Weber et al., 2012). Consequently, suspended sediments and sediment deposition represent distinct exposure pathways that can produce different physiological responses, highlighting the importance of experimental studies that isolate these processes.

The ecological effects of elevated turbidity on corals are complex and context-dependent. Increased turbidity can reduce photosynthetic productivity by attenuating light and limiting the transfer of photosynthetically fixed carbon from symbiotic algae to the coral host (Anthony & Hoegh-Guldberg, 2003; Fabricius, 2005). Conversely, moderate reductions in irradiance have been proposed to lessen photoinhibition during thermal stress, potentially reducing bleaching severity in some coral species (Cacciapaglia & van Woesik, 2016; Sully & van Woesik, 2020). Consequently, turbidity has been reported to both exacerbate and alleviate coral stress depending on sediment characteristics, exposure duration, light availability, water flow, and species-specific sensitivity (Jones et al., 2019; Tuttle & Donahue, 2022). Despite growing recognition that interactions among multiple environmental drivers govern coral responses, relatively few experimental studies have simultaneously evaluated the independent and combined physiological effects of suspended sediments and elevated temperature under controlled laboratory conditions.

The Caribbean reef-building coral, *Orbicella faveolata*, provides an important model species for examining these responses. Historically, species within the *Orbicella* complex were among the dominant framework builders throughout the Caribbean, contributing substantially to reef accretion and structural complexity (Pandolfi & Budd, 2008). However, populations have declined dramatically in recent decades due to disease outbreaks, mass bleaching events, and reduced recruitment (Edmunds, 2015). Understanding the physiological responses of *O. faveolata* to multiple environmental stressors is therefore important for predicting the resilience of Caribbean reef ecosystems under future environmental change. In addition, *O. faveolata* is a primary target for restoration throughout the Florida Reef Tract, making an improved understanding of its physiological responses directly relevant to reef management and restoration.

Coastal management agencies rely on state water-quality criteria and permit-specific compliance monitoring to regulate dredging, shoreline development, and other sediment-generating activities conducted near coral reefs. In Florida, the narrative turbidity criterion specifies that turbidity should not exceed 29 NTU above natural background under most circumstances (Florida Administrative Code Rule 62–302.530), and this benchmark has historically been incorporated into compliance monitoring for dredging and coastal construction projects. However, the physiological basis for interpreting short-term turbidity observations remains limited. Although both suspended sediments and elevated temperature are recognized as important drivers of coral stress, relatively few controlled studies have experimentally isolated their independent effects or evaluated their combined influence across multiple levels of coral biological organization. Consequently, it remains unclear which physiological processes respond first during acute exposure and which metrics provide the most sensitive indicators of sublethal stress. Addressing these knowledge gaps is essential for improving the biological interpretation of coastal water-quality monitoring and informing science-based sediment management.

To address these gaps, we investigated the short-term physiological responses of the Caribbean reef-building coral, *Orbicella faveolata*, to suspended sediment and elevated temperature in controlled laboratory experiments. We first quantified responses across a turbidity gradient (control, 15, 29, and 50 NTU) before evaluating the independent and combined effects of a management-relevant 29 NTU suspended-sediment treatment and elevated temperature in a fully factorial experiment. By experimentally separating suspended sediment from temperature, integrating measurements of photophysiology, metabolism, tissue condition, and calcification, and incorporating a post-exposure recovery period, this study provides one of the first integrated assessments of the hierarchical physiological responses of a Caribbean reef-building coral to these interacting stressors. We hypothesized that suspended sediment would reduce photochemical performance and daytime metabolism through light attenuation, and that elevated temperature would produce broader physiological impairment. We hypothesized that combined exposure would result in greater disruption than either stressor alone. Collectively, these findings provide a mechanistic framework for interpreting acute coral responses to suspended sediment and informing future biological evaluations of sediment-management criteria without directly validating any individual regulatory benchmark.

## Materials and methods

### Coral collection and husbandry

Fragments of *Orbicella faveolata* were obtained from Reef Renewal USA (Tavernier, FL, USA) in four shipments received on August 2, August 16, and August 31, 2023, and February 19, 2024. Fragments originated from offshore coral nurseries maintained by Reef Renewal USA in the Florida Keys. A total of 80 fragments representing eight genotypes were received. Of these, 64 fragments were used in the two experimental phases, and the remaining 16 fragments were destructively sampled to characterize baseline tissue and skeletal condition.

All fragments underwent a standardized 5-week acclimation period under identical laboratory conditions before experimental or baseline sampling to minimize variation associated with shipment and collection history. Upon arrival, coral fragments were placed in a 150-L quarantine system for a total acclimation period of 5 weeks. During this period, fragments were monitored for recovery and for signs of pests, parasites, disease, bleaching, tissue loss, or discoloration. The quarantine system contained the same artificial seawater and was maintained under the same target temperature, salinity, lighting, and water-chemistry conditions as the main holding system described below.

Following quarantine and acclimation, *O. faveolata* fragments were transferred to a 350-L recirculating holding system containing artificial seawater prepared with Red Sea Salt (Red Sea Fish, Tel Aviv, Israel). Sixty-four fragments representing eight genotypes were retained for the experimental treatments, while 16 additional fragments were destructively sampled to characterize baseline tissue and skeletal condition. Water chemistry was monitored regularly, with total alkalinity, calcium, and magnesium measured twice weekly and 25% water changes performed weekly. Target conditions were maintained at approximately 27°C, a salinity of 35, pH 8.1, total alkalinity of approximately 2600 µmol kg^−1^, calcium of 390–420 mg L^−1^, and magnesium of 1250–1350 mg L^−1^.

Artificial lighting was provided by four 180 W LED fixtures (Model MAD180, Wattshine, China) programmed for a 12-h photoperiod from 0600 to 1800 h. Light intensity measured at the base of coral fragments ranged from 150 to 200 µmol photons m^−2^ s^−1^. Nutrient concentrations were monitored using colorimetric test kits (API Saltwater Master Kit, Mars Fishcare, Chalfont, PA, USA; Magnesium, Calcium, and Phosphate Profi Test kits, Salifert, Holland). Weekly measurements of pH (Orion Star A111, Thermo Scientific, Waltham, MA, USA) and total alkalinity (Eco Titrator, Metrohm AG, Herisau, Switzerland) were also conducted to ensure stable water chemistry conditions consistent with those recommended by Enochs et al. (2018) and Manzello et al. (2019).

Corals were fed twice weekly with a mixture of Reef Nutrition Phytoplankton, Bulk Reef Supply Golden Pearls, Polyp Lab Reef-Roids, and Acropower Amino Acids. They were additionally broadcast-fed Reef Nutrition Phytoplankton daily.

### Experimental design

**Fig. 1:**
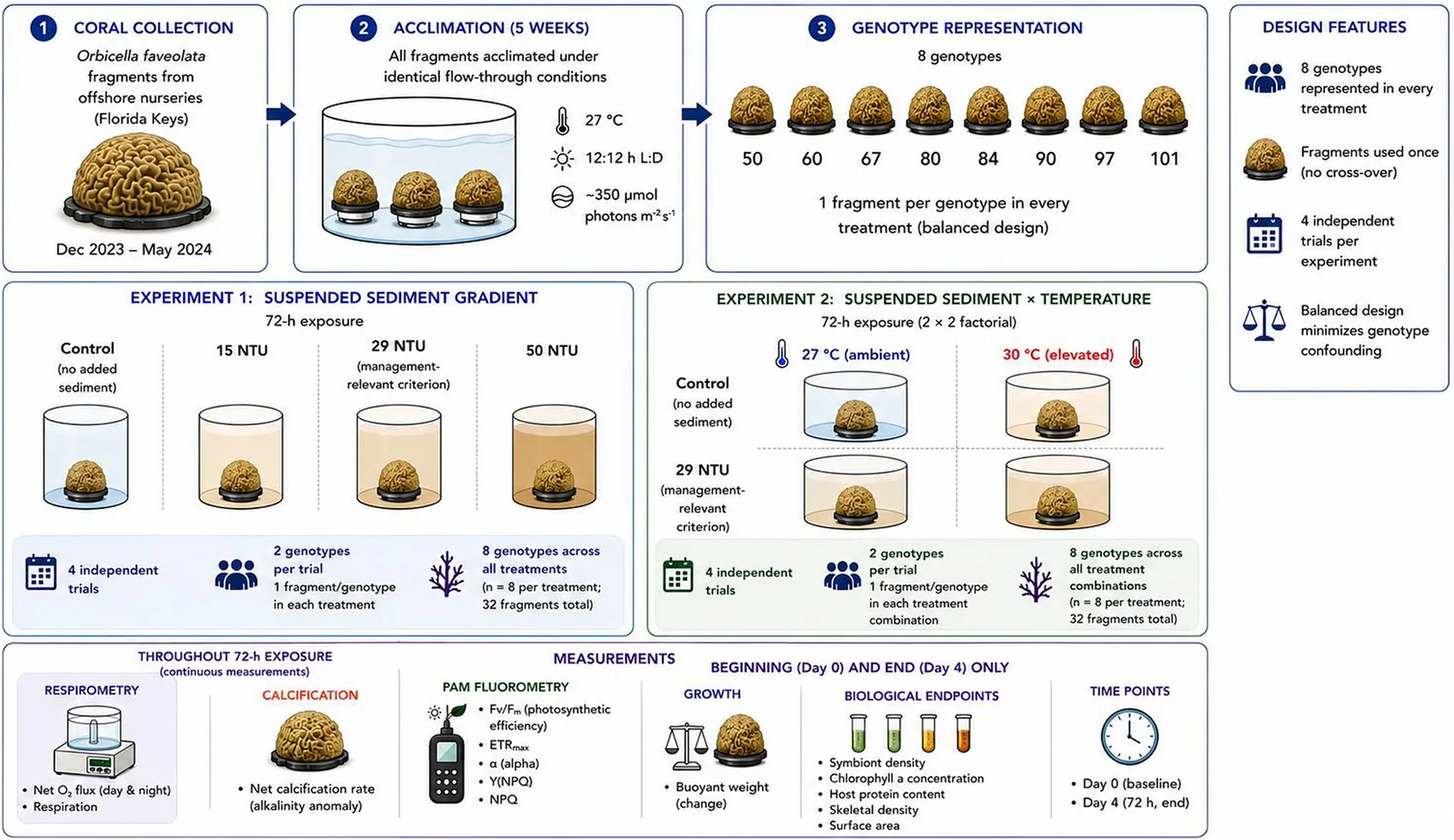
Experimental design schematic illustrating the two sequential 72-h laboratory experiments conducted with *Orbicella faveolata*. Coral fragments representing eight genotypes were collected, acclimated, and distributed evenly among treatments. Experiment 1 evaluated a suspended sediment gradient (control (no added sediment), 15, 29, and 50 NTU). Experiment 2 evaluated the combined effects of suspended sediment (control (no added sediment) or 29 NTU) and temperature (27°C or 30°C) in a factorial design. Physiological responses were assessed using intermittent-flow respirometry, pulse-amplitude modulated (PAM) fluorometry, net calcification, and biological endpoints

This study consisted of two sequential 72h laboratory experiments designed to evaluate the independent and combined effects of suspended sediment and elevated temperature on the physiology of the Caribbean reef-building coral, *Orbicella faveolata*. The first experiment quantified physiological responses across a gradient of management-relevant turbidity levels. In contrast, the second evaluated the combined effects of suspended sediment and elevated temperature using a fully factorial design (Fig. 1). A total of 64 coral fragments representing eight genotypes were used across the two experiments. Thirty-two fragments were used in each experiment, with four fragments from each genotype allocated to each experimental phase. Each fragment was used in only one treatment and one experiment. An additional 16 fragments were destructively sampled before experimentation to characterize baseline biological and skeletal conditions.

Eight coral genotypes (genotype IDs: 50, 60, 67, 80, 84, 90, 97, and 101) were represented throughout the study. Following acclimation, fragments from each genotype were randomly distributed among treatment groups to minimize potential confounding between genotype and experimental treatment. This balanced design ensured that each genotype was represented across all treatments, allowing treatment responses to be evaluated both within and among genotypes while maintaining identical environmental conditions among experimental chambers (Supplemental Table S1).

Each coral fragment was maintained in an independent 500 mL experimental chamber supplied by its own independent 20 L recirculating sump. Thus, no chambers shared treatment water, and each chamber–sump system represented an independent experimental unit. Each system had dedicated circulation, mixing, temperature control, and sediment dosing. Treatment conditions were independently established and maintained within each chamber–sump system, thereby preventing shared-sump effects among coral fragments.

#### Suspended sediment gradient experiment

The suspended sediment gradient experiment evaluated short-term physiological responses across four suspended-sediment treatments, including a control (no added sediment) and target turbidity levels of 15, 29, and 50 NTU. The control treatment received no sediment additions, resulting in a measured background turbidity of 1.19 ± 0.02 NTU (mean ± SE). The selected treatments spanned a gradient from control conditions with no added sediment to target turbidity levels of 15, 29, and 50 NTU. The 29 NTU treatment was included as a management-relevant experimental benchmark because Florida’s narrative turbidity criterion has historically been expressed as 29 NTU above natural background under most circumstances. However, the laboratory treatment represented a target absolute turbidity of 29 NTU rather than a site-specific background plus 29 NTU condition (Florida Administrative Code Rule 62–302.530). It therefore should not be interpreted as a direct experimental replication or validation of the regulatory criterion.

The suspended sediment gradient experiment (Experiment 1) was conducted across four independent trials (Trials 1.2, 1.3, 1.4, and 1.9). Each trial included eight independent chamber–sump systems, with one coral fragment housed in each system. Two genotypes were represented in each trial, and one fragment from each genotype was randomly assigned to one of the four treatments: control (no added sediment), 15, 29, or 50 NTU. Across the four trials, each treatment contained one fragment from each of the eight genotypes, resulting in eight independent experimental units per treatment and 32 fragments overall. This balanced allocation minimized confounding between genotype and treatment and allowed evaluation of treatment responses while accounting for inherent differences among genotypes. Coral fragments were maintained under their assigned treatment conditions for 72 h before post-exposure physiological and biological measurements were completed.

#### Suspended sediment × temperature experiment

The second experiment evaluated the independent and interactive effects of suspended sediment and elevated temperature using a fully factorial design. Coral fragments were exposed to one of four treatment combinations consisting of two temperatures, 27 and 30°C, crossed with two suspended-sediment treatments: a control with no added sediment and a target turbidity of 29 NTU. The 29 NTU treatment was selected as an intermediate treatment from the suspended sediment gradient experiments and as a management-relevant experimental benchmark.

The factorial experiment was conducted across four independent trials (Trials 2.6, 2.7, 2.8, and 2.10). Each trial included eight independent chamber–sump systems, with one coral fragment housed in each system. Two genotypes were represented during each trial, and one fragment from each genotype was randomly assigned to each of the four treatment combinations. Across the four trials, each treatment combination contained one fragment from each of the eight genotypes, resulting in eight independent experimental units per treatment combination and 32 fragments overall. Temperature in the elevated temperature treatments was gradually increased from 27 to 30°C over approximately 12h during the overnight chamber-acclimation period and was then maintained at 30°C throughout the 72h exposure.

### Experimental timeline and sampling protocol

All experimental trials followed an identical 5 day protocol consisting of baseline physiological measurements (day 0), a 72h treatment exposure (days 1–3), and post-exposure physiological measurements and tissue sampling (day 4) (Table 1). This standardized workflow ensured consistent sampling intervals across all experiments while enabling direct comparisons of pre- and post-exposure physiological responses.

**Table 1:** Experimental timeline. Condensed timeline for each trial showing the standardized 5-day workflow

Day	Experimental phase	Measurements
Day 0	Baseline measurements and chamber acclimation	PAM fluorometry, condition photographs, buoyant weight, wet weight, volume displacement; fragments transferred to chambers overnight
Days 1–3	72-h treatment exposure	Sediment and/or temperature treatments maintained; turbidity, temperature, salinity, and TA monitored every 4 h; respirometry monitored continuously
Day 4	Post-exposure measurements and tissue sampling	PAM fluorometry, photographs, buoyant weight, wet weight, volume displacement; tissue subsampling for symbiont density, chlorophyll, protein, and skeletal analyses

#### Day 0—baseline measurements and acclimation

Baseline measurements were collected before treatment exposure to characterize the initial physiological condition of each coral fragment and verify comparable starting conditions among treatment groups. Coral fragments were dark-acclimated for 20 min before pulse-amplitude modulated (PAM) fluorometry measurements were collected using a DIVING-PAM 2.0 fluorometer (Heinz Walz GmbH, Effeltrich, Germany) to assess photosynthetic efficiency (Fv/Fm) and additional photophysiological parameters, including *α*, ETRₘ, and YNPQ. Additional baseline assessments included photographs of the condition, buoyant weight, wet weight, and volume displacement measurements.

Following baseline measurements, fragments were transferred to individual respirometry chambers and allowed to acclimate overnight before treatment initiation. For elevated-temperature treatments, water temperature was gradually increased from 27 to 30°C over approximately 12 h to minimize thermal shock before the start of the experimental exposure (Grottoli et al., 2021).

#### Days 1–3—experimental exposure

Experimental treatments were initiated on day 1 and maintained continuously for 72 h. Suspended sediment concentrations and water quality were monitored throughout the exposure period to ensure treatment stability and consistency among trials (Supplemental Figure S1 & S2).

Turbidity, total alkalinity (TA), temperature, and salinity were measured every 4 h throughout the experiment. Turbidity was quantified using a portable turbidimeter (HACH 2100Q Portable Turbidimeter, HACH Company, Loveland, CO, USA) following U.S. Environmental Protection Agency methods and Florida Department of Environmental Protection Standard Operating Procedures (DEP SOPs; https://floridadep.gov/dear/quality-assurance/content/dep-sops). TA measurements were collected to quantify net calcification using the alkalinity anomaly technique (i.e., Kinsey, 1978; Smith & Kinsey, 1978), while verifying that carbonate chemistry remained consistent throughout the experiment. TA samples were collected from both the experimental system sump and the outflow tubing of each chamber and analyzed within 2 h using an automated titrator (Metrohm Eco Titrator, Metrohm AG, Herisau, Switzerland). Temperature and salinity were measured concurrently using a multiparameter probe (YSI ProQuatro, YSI Inc., Yellow Springs, OH, USA). Measured turbidity remained close to target treatment levels throughout the 72-h exposure, demonstrating stable treatment conditions across all experimental trials (Supplemental Figure S1).

#### Day 4—post-exposure measurements and tissue sampling

Following completion of the 72h exposure, final water quality measurements were collected before coral fragments were removed from the experimental chambers. Fragments were dark-acclimated for 20 min before post-exposure PAM measurements were repeated to quantify changes in photosynthetic efficiency (Fv/Fm) and other photophysiological parameters following the 72h exposure. Condition photographs, buoyant weight, wet weight, and volume displacement measurements were subsequently repeated to quantify treatment-induced changes.

Each coral fragment was then subsampled using a diamond-tipped saw for destructive endpoint analyses. Tissue samples were immediately flash-frozen and stored at −80 °C until subsequent analyses of symbiont density, chlorophyll concentration, host protein content, and skeletal properties. Remaining coral fragments were returned to the holding system to monitor recovery following experimental exposure.

### Sediment characterization

Sediment used in all experiments was collected from Port Everglades, Florida, shipped frozen, and stored at −20 °C until processing. Prior to characterization, sediments were thawed, homogenized, dried at 80°C for 48 h, and ground using a cryogenic mill (6875 Freezer/Mill, Cole-Parmer, Vernon Hills, IL, USA) to produce a homogeneous sediment matrix for subsequent analyses.

Sediment composition was quantified by sequential combustion to determine moisture, carbonate, and terrigenous fractions. Samples were combusted at 100°C for 10 h, 500°C for 12 h, and 1000°C for 2 h, following standard gravimetric procedures. Grain size distribution was determined from 30 g subsamples that were wet-sieved into three fractions (< 63 µm, 63–125 µm, and > 125 µm). Organic material was removed by digestion with 30% hydrogen peroxide prior to particle size analysis using a laser diffraction particle size analyzer (Beckman-Coulter LS13 320, Beckman Coulter Inc., Brea, CA, USA).

Prior to each experimental trial, sediment was cryomilled to achieve a consistent particle-size distribution and ensure homogeneous suspended-sediment characteristics across treatments. Sediment characteristics are summarized in Supplemental Table S2.

### Experimental treatments

#### Suspended sediment gradient experiments (Experiment 1)

A sediment calibration curve was developed to determine the mass of suspended sediment required to achieve target turbidity levels in seawater. Sediment was incrementally added to 1 L of filtered seawater, and turbidity was measured following each addition to generate a linear calibration equation relating sediment concentration (g L^−1^) to turbidity (NTU) (*R*^2^ = 0.9959). This relationship was used to calculate the sediment mass required to achieve the target treatments of 15, 29, and 50 NTU.

Before each experiment, sediment was added to the experimental sump approximately 1 h before treatment initiation to allow turbidity to stabilize. Suspended sediment concentrations were maintained using overhead stirrers (300 rpm) and automated dosing pumps (Jecod Co., Ltd.) that continuously delivered a well-mixed sediment stock solution (55 g sediment in 5 L seawater, 25 ppt). Dosing volumes were adjusted as needed to maintain target turbidity for each sump while preserving consistent salinity among treatments. Turbidity was monitored throughout the 72h exposure period, and sediment additions were adjusted when necessary to maintain target treatment levels. Measured turbidity varied by less than ± 5 NTU from target values throughout the experiment (Supplemental Figure S1). The 72h exposure duration was selected to quantify early physiological responses to suspended sediment while minimizing the effects of longer-term acclimation and chronic stress.

#### Suspended sediment × temperature experiment (Experiment 2)

Elevated temperature treatments were maintained using two titanium aquarium heaters (200 W) connected to recirculating sump-water baths. Temperatures were gradually increased from 27 to 30°C during the overnight acclimation period and were maintained throughout the 72h exposure. Chamber temperature was verified using an infrared thermometer (Hyper Tough Model 1504 V). In contrast, sump temperature and salinity were measured using a handheld multiparameter probe (YSI ProQuatro, YSI Inc., Yellow Springs, OH, USA). Continuous temperature logging was performed using HOBO Pendant temperature loggers (Onset Computer Corporation, Bourne, MA, USA) placed within each sump to verify treatment stability throughout the experiment. Temperature remained within ± 0.2°C of target values throughout the experiment (Supplemental Figure S2).

To maintain water quality, all treatments received daily 5 L water exchanges using seawater pre-adjusted to the appropriate suspended sediment treatment. Water exchanges minimized algal growth while maintaining consistent salinity and target turbidity throughout the experiment.

### Experimental respirometry system

Holobiont metabolic oxygen flux was quantified using a custom-built intermittent-flow respirometry system consisting of eight independent chamber–sump units. Each unit contained one 500 mL acrylic respirometry chamber (Loligo Systems, Denmark) connected to its own 20 L recirculating sump and optical oxygen sensor (Witrox-4, Loligo Systems). Each chamber was supplied only with water from its associated sump using an external circulation pump (Eheim Universal 300), and no water was shared among experimental units. Internal magnetic stirring maintained homogeneous mixing within each chamber and helped keep sediment in suspension. Coral fragments were elevated on wire pedestals to prevent contact with the stir bars and minimize sediment accumulation on tissue surfaces.

Each sump was mixed continuously using a dedicated overhead stirrer and aerated with an airstone to maintain sediment suspension and oxygen saturation. Suspended sediment additions, temperature control, water exchanges, and water-quality measurements were conducted independently for each chamber–sump system. Artificial irradiance was provided by LED fixtures (A80 Tuna Blue, Kessil, Richmond, CA, USA) at 150–200 µmol photons m^−2^ s^−1^.

### Respirometry measurements

Holobiont metabolic oxygen flux (MO₂; mg O₂ g^−1^ h^−1^) was measured using intermittent-flow respirometry, which periodically flushes chambers with fresh seawater to prevent the accumulation of metabolic waste products while maintaining oxygen concentrations above hypoxic levels (> 80% saturation) (Svendsen et al., 2016). Because oxygen production and consumption respond rapidly to environmental perturbation, intermittent-flow respirometry provides a sensitive measure of sublethal physiological stress before detectable changes occur in tissue biomass or skeletal growth.

Dissolved oxygen concentrations were recorded using polymer optical fiber sensors connected to a Witrox-4 oxygen transmitter (Loligo Systems). Sensors were calibrated before each experimental trial using a two-point calibration consisting of oxygen-saturated seawater (100% saturation) and sodium sulfite solution (0% saturation; 10 g Na₂SO₃ in 500 mL water). Each oxygen sensor was paired with a temperature probe positioned within the corresponding sump to continuously record water temperature for oxygen solubility calculations.

Each respirometry cycle consisted of a 2-min flush period, a 2-min equilibration period, and a 56-min measurement period during which oxygen concentrations were continuously recorded while chambers remained closed (Svendsen et al., 2016). Oxygen saturation remained above 80% throughout all measurement periods.

### Respirometry data processing

Dissolved oxygen concentrations were recorded at 1 Hz throughout each 72h experiment (Loligo Autoresp 2.0 software). To eliminate artifacts associated with chamber flushing, the first and last 2 min of each hourly measurement cycle were excluded from analysis. Rolling linear regressions were then applied to oxygen data collected between minutes 2 and 58 of each measurement cycle using the R package *RespR* (Harianto et al., 2019), generating hourly estimates of metabolic oxygen flux for each chamber. Quality control procedures followed the recommendations of Harianto et al. (2019). Oxygen traces were visually inspected for deviations from linearity caused by air bubbles, incomplete flushing, or sensor interference. Measurement periods exhibiting these artifacts, as well as any traces with a coefficient of determination (*R*^2^) < 0.80, were excluded from subsequent analyses.

Background microbial respiration was quantified using empty respirometry chambers following each experimental trial and was used to correct oxygen flux measurements not caused by the coral. Continuous empty-chamber measurements were not feasible because all chambers were occupied during experimental exposures.

### Calculation of metabolic oxygen flux

Mass-specific holobiont metabolic oxygen flux (MO₂; mg O₂ g^−1^ h^−1^) was calculated as:

$$M{O}_{2}=\left(\frac{\Delta C}{\Delta t}\right)\frac{V}{M}$$

where Δ*C*/Δ*t* is the rate of change in dissolved oxygen concentration during the measurement period, *V* is the effective chamber volume corrected for coral displacement and recirculation tubing volume, and *M* is the wet mass of the coral fragment measured before experimental exposure. Oxygen flux values were expressed as mg O₂ g^−1^ h^−1^. Positive daytime values represent net oxygen production, whereas negative values represent oxygen consumption.

### Net calcification (G_net_)

Net calcification (*G*ₙₑₜ) was calculated using the total alkalinity (TA) anomaly technique (Kinsey, 1978; Smith & Kinsey, 1978) based on changes in TA between the chamber and sump following a 56-min incubation. TA samples were collected at two time points: initially from the sump before the flush and subsequently from the water exiting the chamber during the flush. After collection, samples were brought to room temperature (25°C) in a water bath, weighed using an analytical balance (VWR-224AC), and analyzed by potentiometric titration (Metrohm Eco Titrator). Duplicate water samples from each chamber and sump were analyzed and averaged. If duplicate measurements differed by more than 5 µmol kg^−1^, a third sample was analyzed. Sample pH was verified using a benchtop pH meter (Thermo Scientific). Net calcification rates (µmol CaCO₃ g buoyant wt^−1^ h^−1^) were calculated from changes in total alkalinity (ΔTA) using the equation described by McNicholl and Koch (2021) and normalized to the initial buoyant weight of each coral fragment following Jokiel et al. (1978). Initial buoyant weight was measured for all coral fragments prior to experimental exposure. Coral fragments did not differ significantly in initial buoyant weight among treatment groups, confirming that fragment size was comparable before the experiment.

### Pulse-amplitude modulated (PAM) photophysiology

Photosynthetic performance of the algal symbionts was quantified using pulse-amplitude modulated (PAM) fluorometry (DIVING-PAM 2.0; Heinz Walz GmbH, Effeltrich, Germany). PAM fluorometry provides a sensitive, non-destructive assessment of symbiont photophysiology and was used to detect early changes in photosynthetic performance resulting from suspended sediment and thermal stress before measurable changes in coral tissue condition or growth. Measurements were collected immediately before treatment exposure (baseline), immediately following the 72-h exposure, and during the post-experimental recovery period.

Prior to each measurement, coral fragments were dark-acclimated for at least 20 min to minimize photochemical quenching and permit accurate determination of the maximum quantum yield of photosystem II (Fv/Fm) (Buxton et al., 2012; Maxwell & Johnson, 2000). All measurements were conducted at the same time of day to minimize diel variation in symbiont photophysiology.

Whole *Orbicella faveolata* fragments were measured at two locations on each colony to account for spatial heterogeneity in symbiont activity. Fragments designated for post-experimental recovery were measured once following destructive subsampling because only half of the colony remained.

The fiber-optic probe was positioned approximately 5 mm above the coral surface using a fixed spacer to maintain a consistent measurement distance among samples. Instrument gain (3–4) and measuring-light intensity (5–7) were adjusted for each fragment to maintain initial fluorescence (*F*₀) values between 300 and 500 relative fluorescence units (RFU), ensuring adequate signal quality despite natural variation in coral pigmentation, tissue characteristics, and symbiont density.

Rapid light curves were generated to quantify maximum quantum yield of photosystem II (Fv/Fm), maximum electron transport rate (ETRₘ), the initial slope of the rapid light curve (*α*), and non-photochemical quenching yield (YNPQ). Together, these parameters characterize photochemical efficiency, maximum photosynthetic capacity, light-use efficiency under low irradiance, and photoprotective energy dissipation, providing a complementary assessment of symbiont responses to experimental stress.

### Biological endpoints

To determine whether early physiological responses were accompanied by changes in holobiont condition and skeletal properties, destructive biological endpoints were quantified following each experimental trial. Measurements included Symbiodiniaceae abundance (i.e., symbiont density), chlorophyll concentrations (chlorophyll *a*, chlorophyll *c*₂, and total chlorophyll), host protein concentration, skeletal density, and skeletal surface area. Together, these complementary metrics provided an integrated assessment of symbiont condition, host tissue biomass, and skeletal properties following exposure to suspended sediment and elevated temperature.

Coral tissue was removed from each fragment using an airbrush (Paasche Airbrush Co., Kenosha, WI, USA) with phosphate-buffered saline (PBS). The resulting tissue slurry (20 mL) was homogenized by sonication for 20 s (Qsonica LLC, Newtown, CT, USA), thoroughly mixed, and subsampled for Symbiodiniaceae, chlorophyll, and protein analyses.

Symbiodiniaceae abundance was quantified by hemocytometer counts (Bright-Line, Hausser Scientific, Horsham, PA, USA) using an ICC50W microscope (Leica Microsystems Inc., Deerfield, IL, USA). Chlorophyll *a* and chlorophyll *c*₂ concentrations were determined spectrophotometrically (SpectraMax M3, Molecular Devices, San Jose, CA, USA) following acetone extraction of algal pigments and calculated using the equations of Jeffrey and Humphrey (1975). Host protein concentration was quantified spectrophotometrically following using absorbance at 235 and 280 nm and calculated as (*A*₂₃₅ − *A*₂₈₀)/2.51.

Following tissue removal, coral skeletons were cleaned in a 10% bleach solution, dried at 60°C for 4 h, and weighed to determine skeletal dry mass. Skeletal density (g cm^−3^) was calculated by dividing skeletal dry mass by skeletal volume determined using the water displacement method. Three-dimensional scans of each skeleton were generated using an Einscan-SE 3D scanner (Shining 3D, Hangzhou, China), and skeletal surface area was calculated using MeshLab software.

All biological endpoints were corrected for dilution factors, where applicable. Symbiodiniaceae abundance, chlorophyll concentrations, and host protein concentration were standardized to skeletal surface area (cm^2^). Final response variables included Symbiodiniaceae abundance (cells × 10^6^ cm^−2^), chlorophyll concentrations (µg cm^−2^), host protein concentration (mg cm^−2^), skeletal density (g cm^−3^), and skeletal surface area (cm^2^).

### Statistical approach

All statistical analyses were conducted in R (version 4.4.1). Linear mixed-effects models were fit using the *lme4* package (Bates et al., 2015), with denominator degrees of freedom estimated using *lmerTest* (Kuznetsova et al., 2017). Estimated marginal means were calculated using the *emmeans* package (Lenth, 2024). Model assumptions were evaluated by visually inspecting residual-versus-fitted and quantile–quantile plots for homogeneity of variance and normality of residuals, respectively. Statistical significance was evaluated at *α* = 0.05.

For the suspended sediment gradient experiments, biological endpoints were analyzed using linear models with turbidity and genotype included as fixed effects. For the suspended-sediment × temperature experiments, biological endpoints were analyzed using linear models with turbidity, temperature, the turbidity × temperature interaction, and genotype as fixed effects.

Photophysiological responses were quantified as the change between pre-exposure (day 0) and post-exposure (day 4) measurements (*Δ* = day 4 − day 0) for maximum quantum yield (Fv/Fm), maximum electron transport rate (ETRₘ), the initial slope of the rapid light curve (*α*), and non-photochemical quenching (YNPQ). These response variables were analyzed using linear models with turbidity, temperature, the turbidity × temperature interaction, and genotype included as fixed effects.

The individual chamber–sump system was treated as the experimental unit because each coral fragment was maintained in a separate chamber supplied by an independent sump with independently controlled treatment conditions. No experimental units shared treatment water. Genotype was included as a fixed blocking factor to account for inherent physiological differences among genotypes, and trial was included as a random effect to account for temporal and operational variation among the four independently conducted trials.

Net calcification and holobiont oxygen flux (MO₂) were analyzed using linear mixed-effects models. For the suspended sediment gradient experiments, turbidity and genotype were included as fixed effects. For the suspended sediment × temperature experiment, turbidity, temperature, the turbidity × temperature interaction (for net calcification), genotype, and time of day (for MO₂) were included as fixed effects. Experimental day and chamber were included as random effects to account for variation among experimental runs and chamber-specific conditions. Oxygen-flux measurements were standardized to coral skeletal mass, and measurements in which oxygen saturation fell below 80% were excluded, following Svendsen et al. (2016).

For linear models, the significance of model coefficients was evaluated using *t*-tests from the model summaries. For linear mixed-effects models, significance was assessed using the *lmerTest* package. When significant treatment effects or interactions were detected, post hoc pairwise comparisons were performed using estimated marginal means with Tukey adjustment (*emmeans* package). Results are presented as mean ± standard error (SE).

## Results

Coral physiological responses were assessed across multiple levels of biological organization, progressing from photophysiological performance (Fv/Fm, ETRmax, YNPQ, and *α*) to metabolic function (oxygen flux; MO₂), tissue condition (Symbiodiniaceae density, chlorophyll concentration, and host protein content), and skeletal growth (net calcification; *G*_net_).

### Sediment characterization

Sediment collected from Port Everglades, Florida, consisted primarily of terrigenous material, comprising an average of 65.32 ± 1.48% of the total sample, followed by carbonate material (31.07 ± 1.89%), organic matter (3.13 ± 0.41%), and moisture (0.48 ± 0.09%) (Supplemental Table S2). Prior to milling, the sediment was dominated by coarse sand (500–2,000 µm; 67.43 ± 3.51%), followed by medium sand (250–500 µm; 25.25 ± 1.44%), while fine sand (63–250 µm; 2.68 ± 1.35%) and silt/clay (< 63 µm; 0.25 ± 0.08%) represented only a small proportion of the sediment. Cryomilling produced a more homogeneous particle-size distribution by reducing the abundance of large particles and increasing the proportion of fine suspended particles used in the experimental exposures. Following milling, 20.23% of particles were < 63 µm, 24.92% were between 63 and 125 µm, 51.54% were between 250 and 500 µm, and only 3.32% remained at approximately 1000 µm, with no particles > 2000 µm (Supplemental Table S2).

### Experimental conditions

#### Suspended sediment gradient experiments

Target turbidity treatments were control (no sediment added), 15, 29, and 50 NTU. Measured turbidity closely reflected the intended treatment gradient, averaging 1.12 ± 0.32 NTU, 15.76 ± 2.82 NTU, 28.79 ± 4.31 NTU, and 52.02 ± 5.22 NTU, respectively (Supplemental Table S3). Temperature remained stable throughout the experiments, averaging 26.71 ± 0.22°C across treatments. Salinity ranged from 35.26 to 36.63, while dissolved oxygen concentrations generally ranged from 6.15 to 6.72 mg L^−1^, indicating stable, well-oxygenated conditions. One chamber exhibited greater variability in dissolved oxygen but remained well oxygenated overall (Supplemental Table S3).

#### Suspended sediment × temperature experiment

The suspended sediment × temperature experiment combined two target turbidity levels (i.e., control (no sediment added) and 29 NTU) with ambient and elevated temperature treatments of 27°C and 30°C, respectively. No-added-sediment chambers maintained low turbidity, averaging 1.25 ± 0.47 NTU, whereas suspended-sediment chambers averaged 29.99 ± 4.59 NTU, closely approximating the intended 29 NTU treatment (Supplemental Table S3). Ambient and elevated temperature treatments averaged 27.25 ± 0.24°C and 29.99 ± 0.25°C, respectively. Salinity ranged from 34.02 to 36.38, and dissolved oxygen concentrations ranged from 5.95 to 6.54 mg L^−1^, indicating generally stable water-quality conditions across treatment combinations (Supplemental Table S3).

### Photophysiological responses

#### Suspended sediment gradient experiments

**Fig. 2:**
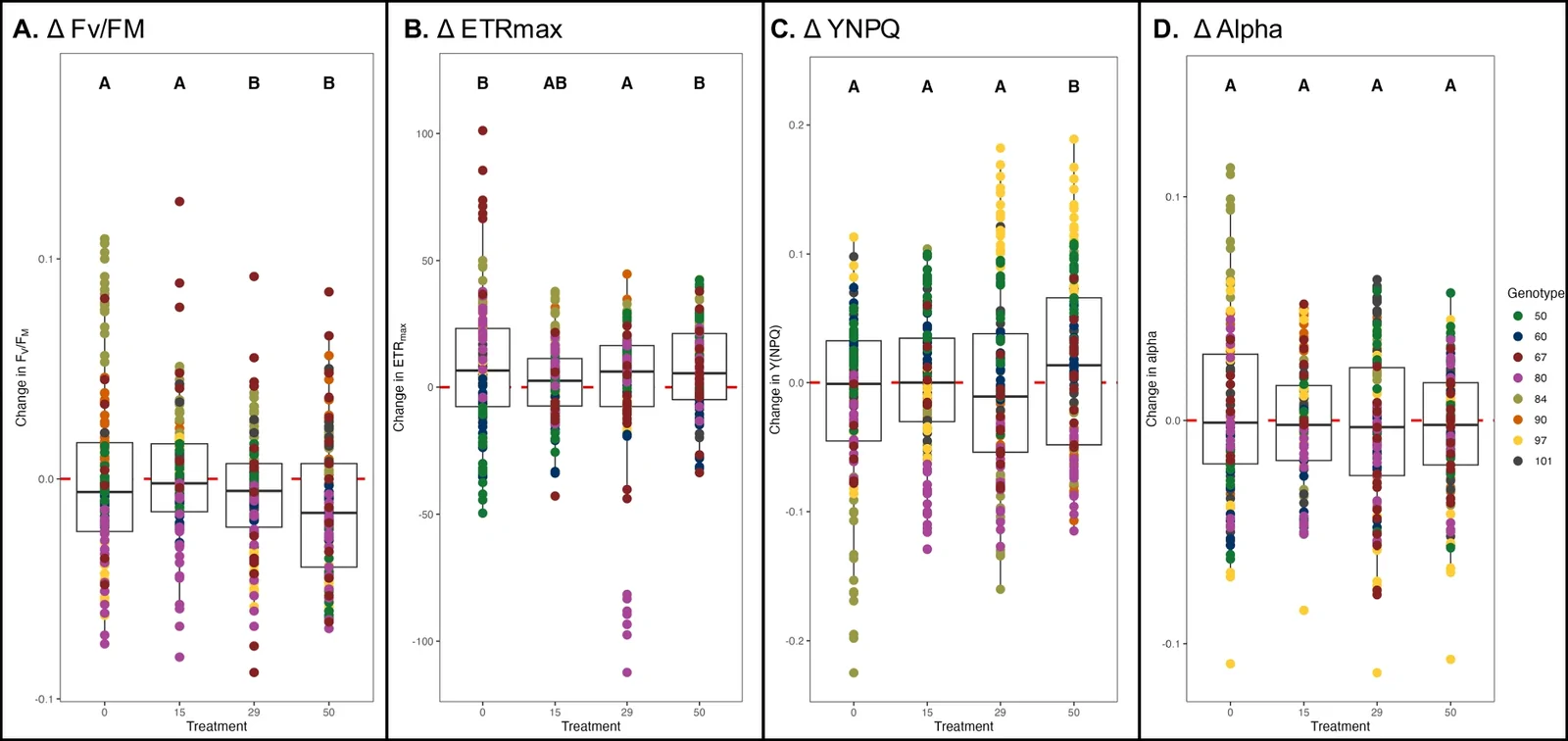
Changes in photophysiological parameters of *Orbicella faveolata* following 72h exposure to suspended sediment treatments (control, 15, 29, and 50 NTU). Responses are expressed as the change from pre-exposure values (*Δ*) for maximum photochemical efficiency (ΔFv/Fm; **A**), maximum electron transport rate (ΔETmax; **B**) regulated non-photochemical energy dissipation (ΔYNPQ; **C**), and the initial slope of the rapid light curve (Δ*α*; **D**). Boxplots represent the median, interquartile range, and 1.5 × interquartile range, with individual colonies shown as colored points corresponding to genotype. The dashed red line indicates no change from pre-exposure values (*Δ* = 0). Treatments sharing the same uppercase letter are not significantly different (Tukey-adjusted, *p* > 0.05)

Photophysiological responses varied among suspended sediment treatments during the suspended sediment gradient experiments (Experiment 1; Fig. 2; Table S5). Maximum relative electron transport rate (ΔETRmax) differed significantly among treatments (*p* = 0.004), with post hoc comparisons indicating lower values at 29 NTU than at both the control (no sediment added; *p* = 0.005) and 50 NTU (*p* = 0.023). Maximum quantum yield (ΔFv/Fm) declined significantly with suspended sediment, with lower values at both 29 NTU (*p* = 0.026) and 50 NTU (*p* < 0.001) relative to the control. In contrast, regulated non-photochemical energy dissipation (ΔYNPQ) increased under the highest suspended sediment treatment, with significantly greater values at 50 NTU than the control (*p* < 0.001), 15 NTU (*p* = 0.026), and 29 NTU (*p* = 0.009). The initial slope of the rapid light curve (*α*) was not significantly affected by suspended sediment (*p* = 0.055). After accounting for suspended sediment treatment, significant genotype effects were detected for *α*, ΔETRmax, and ΔFv/Fm, indicating substantial baseline variation in photophysiological performance among coral genotypes.

#### Suspended sediment × temperature experiment

**Fig. 3:**
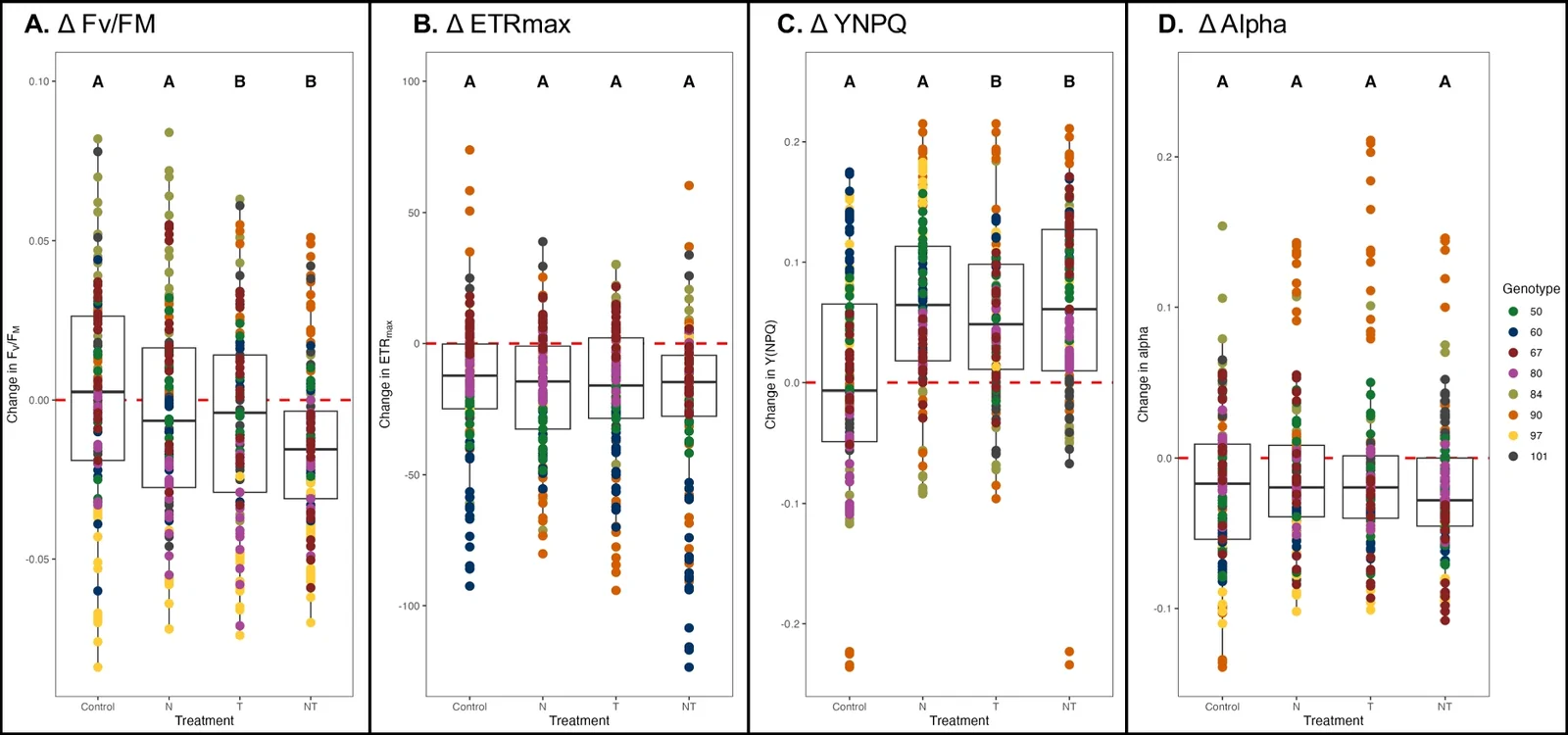
Changes in photophysiological parameters of *Orbicella faveolata* following 72h exposure to the factorial suspended sediment × temperature treatments: control (27 °C, no added sediment), elevated suspended sediment (N; 29 NTU), elevated temperature (T; 30 °C), and combined suspended sediment and elevated temperature (NT; 29 NTU + 30 °C). Responses are expressed as the change from pre-exposure values (*Δ*) for maximum photochemical efficiency (ΔFv/Fm; **A**), maximum electron transport rate (ΔETmax; **B**), regulated non-photochemical energy dissipation (ΔYNPQ; **C**), and the initial slope of the rapid light curve (Δ*α*; **D**). Boxplots represent the median, interquartile range, and 1.5 × interquartile range, with individual colonies shown as colored points corresponding to genotype. The dashed red line indicates no change from pre-exposure values (*Δ* = 0). Treatments sharing the same uppercase letter are not significantly different (Tukey-adjusted, *p* > 0.05)

Photophysiological responses differed among suspended sediment and temperature treatments during experiments (Fig. 3; Table S6). The initial slope of the rapid light curve (*α*) and maximum relative electron transport rate ΔETRmax were not significantly affected by suspended sediment, elevated temperature, or their interaction (all *p* ≥ 0.12). In contrast, elevated temperature significantly reduced maximum quantum yield (ΔFv/Fm; *p* = 0.024), whereas suspended sediment had no effect and no interaction was detected. Regulated non-photochemical energy dissipation (ΔYNPQ) was significantly affected by suspended sediment (*p* = 0.016), elevated temperature (*p* < 0.001), and their interaction (*p* = 0.019). Post hoc comparisons indicated significantly greater ΔYNPQ at 30°C than at 27°C (*p* < 0.001). Significant genotype effects were observed for *α*, ΔETRmax, and ΔFv/Fm but not for ΔYNPQ.

#### Post-treatment recovery of photophysiology

Recovery of maximum photochemical efficiency (Fv/Fm) was evaluated 4 weeks after experimental exposure by comparing post-recovery values with pre-exposure measurements (Supplemental Figure S3). No significant differences in ΔFv/Fm were detected among treatments in either the suspended sediment gradient or the suspended sediment × temperature experiments. Across both experiments, Fv/Fm values had returned to levels comparable to those measured before exposure within the 4-week recovery period, although variability among genotypes was evident.

### Holobiont oxygen flux (MO_2_)

#### Suspended sediment gradient experiments

**Fig. 4:**
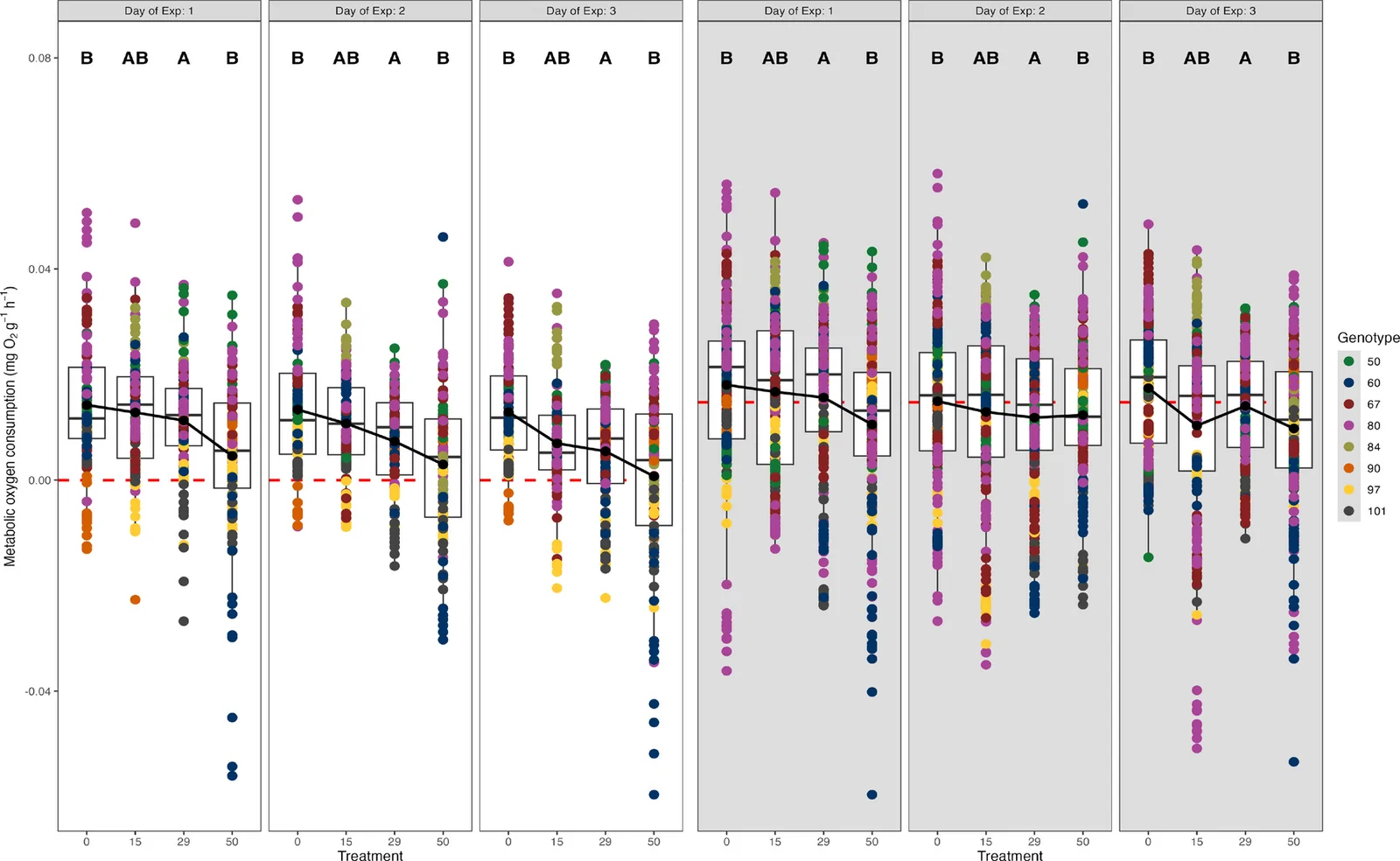
Daytime and nighttime holobiont oxygen flux (MO₂) of *Orbicella faveolata* across suspended sediment treatments (control, 15, 29, and 50 NTU) over the three experimental days. Panels show measurements separated by time of day and day of exposure. Colored points represent individual coral fragments and correspond to genotype. Boxplots show the median and interquartile range, while black points and connecting lines represent treatment means ± SE. The dashed red line indicates zero net oxygen flux. Treatments sharing the same uppercase letter are not significantly different (Tukey-adjusted, *p* > 0.05)

Holobiont oxygen flux (MO₂) remained relatively consistent across suspended sediment treatments during the experiments (Fig. 4), although mean respiration exhibited a slight declining trend with increasing suspended sediment concentration. The mixed-effects model identified a reduction in MO₂ at the highest suspended sediment treatment (50 NTU) relative to the reference level; however, no pairwise differences among turbidity treatments remained significant following Tukey adjustment (all adjusted *p* ≥ 0.052; Table S5). Time of day did not significantly influence MO₂ (*p* = 0.890). After accounting for treatment effects, significant genotype differences remained in holobiont oxygen flux, indicating substantial baseline physiological variation among coral genotypes.

#### Suspended sediment × temperature experiment

**Fig. 5:**
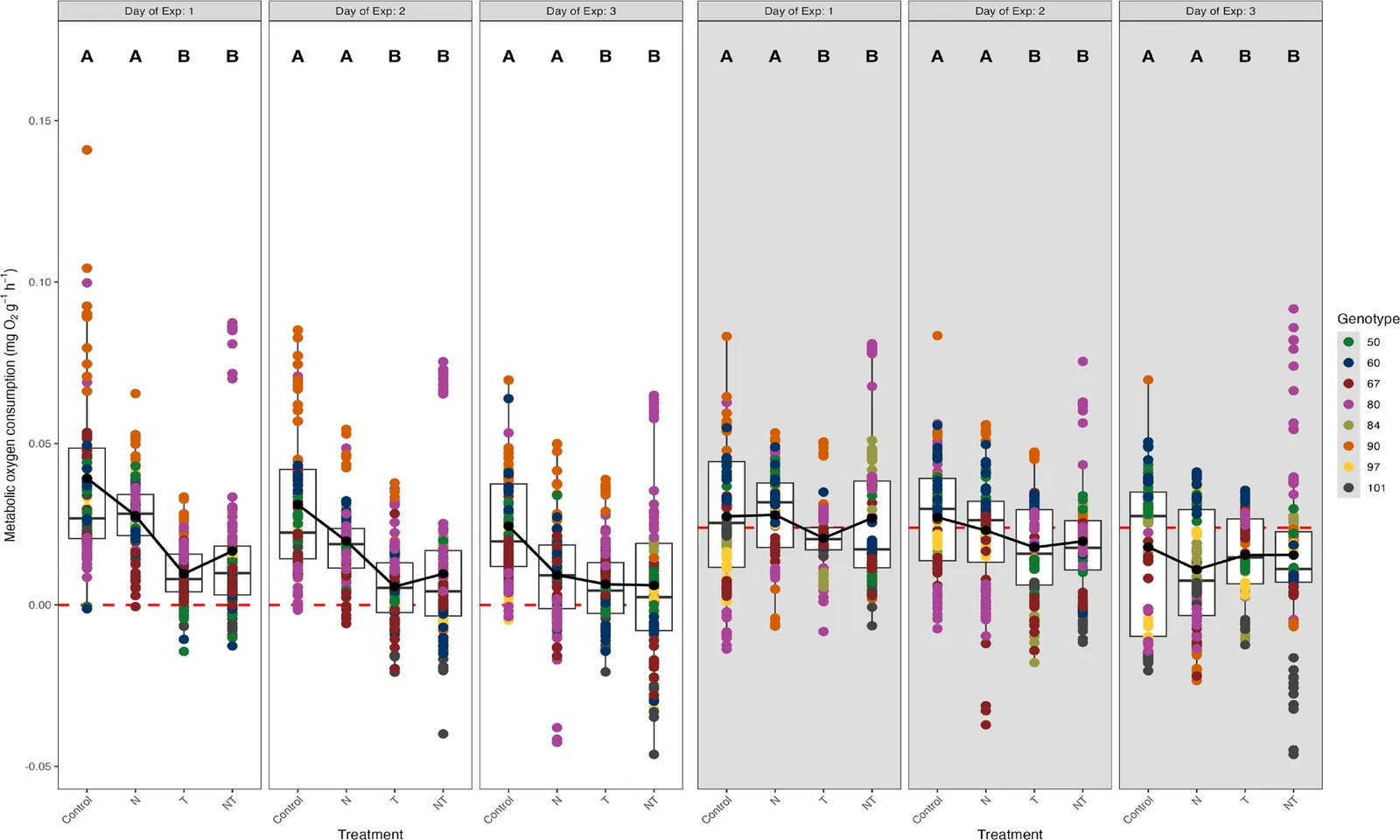
Daytime and nighttime holobiont oxygen flux (MO₂) of *Orbicella faveolata* across the suspended sediment × temperature treatments over the three experimental days. Treatments were control (27°C, no added sediment), elevated suspended sediment (N; 29 NTU), elevated temperature (T; 30°C), and combined suspended sediment and elevated temperature (NT; 29 NTU + 30°C). Panels show measurements separated by time of day and experimental day. Colored points represent individual coral fragments and correspond to genotype. Boxplots show the median and interquartile range, while black points and connecting lines represent treatment means ± SE. The dashed red line indicates zero net oxygen flux. Treatments sharing the same uppercase letter are not significantly different (Tukey-adjusted, *p* > 0.05)

Holobiont oxygen flux was significantly affected by temperature but not by suspended sediment or the turbidity × temperature interaction (Table S6). Mean MO₂ (± SE) was lower in corals maintained at 30°C than at 27°C (Fig. 5), and post hoc comparisons confirmed significantly lower respiration under elevated temperature (adjusted *p* = 0.034). In contrast, respiration did not differ between suspended sediment treatments (adjusted *p* = 0.391), and no significant turbidity × temperature interaction was detected (*p* = 0.110). As expected, mean daytime MO₂ was substantially greater than nighttime MO₂ (adjusted *p* < 0.001; Fig. 5), reflecting diel changes in holobiont metabolism. Significant genotype effects were also detected.

### Biological endpoints

#### Suspended sediment gradient experiments

Indicators of coral tissue condition, including Symbiodiniaceae density, chlorophyll concentrations, and soluble protein content, remained largely stable across suspended sediment treatments (Figure S4; Table S5). Total chlorophyll concentration and chlorophyll per symbiont cell were not significantly affected by suspended sediment (all *p* ≥ 0.087). Chlorophyll *a* was significantly lower only in corals exposed to the highest suspended sediment treatment (50 NTU; *p* = 0.007), whereas soluble protein concentration and Symbiodiniaceae density were significantly lower only in the 15 NTU treatment (*p* = 0.007 and *p* = 0.023, respectively), with no significant differences detected at higher suspended sediment concentrations. These isolated responses did not exhibit a consistent dose-dependent pattern, suggesting that short-term exposure to suspended sediment had minimal effects on overall tissue condition. Even after accounting for experimental treatments, significant genotype effects were detected for chlorophyll *a*, chlorophyll per symbiont cell, soluble protein concentration, and Symbiodiniaceae density, indicating substantial baseline physiological variation among coral genotypes.

#### Suspended sediment × temperature experiment

Indicators of coral tissue condition remained stable throughout the suspended sediment × temperature experiments (Figure S5; Table S6). Neither suspended sediment, elevated temperature, nor their interaction significantly affected total chlorophyll concentration, chlorophyll *a*, chlorophyll per symbiont cell, soluble protein concentration, or Symbiodiniaceae density (all *p* ≥ 0.21). Significant genotype effects were observed for total chlorophyll, chlorophyll *a*, chlorophyll per symbiont cell, soluble protein concentration, and Symbiodiniaceae density, reflecting inherent physiological differences among coral genotypes rather than treatment responses. Collectively, these results indicate that tissue biomass and symbiont characteristics were maintained despite exposure to suspended sediment, elevated temperature, and their combination over the experimental period.

### Net calcification (G_net_)

Initial buoyant weight did not differ significantly among turbidity treatments in suspended sediment gradient experiments (Experiment 1; one-way ANOVA, *F*₃,₂₈ = 1.95, *p* = 0.15), between turbidity treatments in suspended sediment × temperature experiments (Experiment 2; *p* = 0.87), or between ambient (27°C) and elevated (30°C) temperature treatments in suspended sediment x temperature experiments (*p* = 0.90). These results indicate that coral fragment size was comparable among treatment groups before experimental exposure.

#### Suspended sediment gradient experiments

Net calcification remained relatively stable across suspended sediment treatments during the suspended sediment experiments, with no significant effect of turbidity on *G*_net_ (all *p* ≥ 0.334; Figure S5, Table S5). Mean *G*_net_ (± SE) varied among individual colonies and experimental trials but showed no consistent response to increasing suspended sediment concentrations (Figure S5). Although corals exposed to 50 NTU exhibited a slight numerical reduction in mean net calcification relative to the control, differences among treatments were not statistically significant. Significant genotype effects were detected, with genotypes 90 and 97 exhibiting greater net calcification than the reference genotype (*p* = 0.030 and *p* = 0.033), indicating substantial baseline variation among coral genotypes.

#### Suspended sediment × temperature experiment

Net calcification also remained stable during the suspended sediment × temperature experiment, with no significant effects of suspended sediment, elevated temperature, or their interaction on *G*_net_ (all *p* ≥ 0.635; Figure S5, Table S6). Mean *G*_net_ (± SE) was similar among all treatment combinations (Figure S5), indicating that neither elevated temperature nor suspended sediment measurably altered calcification over the 72-h exposure period. After accounting for suspended sediment and temperature treatments, significant genotype effects remained for net calcification, with genotypes 67, 80, 90, and 101 exhibiting higher rates than the reference genotype (all *p* ≤ 0.025). This result highlights substantial inherent variation in calcification among coral genotypes.

### Integrated physiological responses to suspended sediment and elevated temperature

Across both experimental phases, photophysiological metrics were the most responsive physiological endpoints (Table 2). During the suspended sediment gradient experiment, suspended sediment significantly affected ΔETRmax, ΔFv/Fm, and ΔYNPQ, whereas *α*, gross net calcification, and most biological endpoints showed little response. Respiration exhibited only modest treatment effects.

**Table 2:** Summary of statistically significant physiological responses of *Orbicella faveolata* to suspended sediment and elevated temperature. A checkmark (✓) denotes a significant effect (*p* < 0.05) of the corresponding factor, whereas a dash (—) denotes no significant effect. Experiment 1 consisted of a suspended sediment gradient (control, 15, 29, and 50 NTU), and Experiment 2 consisted of a factorial experiment testing suspended sediment (control and 29 NTU), temperature (27 and 30 °C), and their interaction. The “Pattern” column provides a brief description of the direction or treatment level(s) associated with significant responses

Endpoint	Suspended sediment experiments	Suspended sediment × temperature experiments			Pattern
	Sediment	Sediment	Temperature	Interaction	
ΔETRmax	✓	—	—	—	Lowest at 29 NTU
ΔFv/Fm	✓	—	✓	—	Declined with sediment and warming
ΔYNPQ	✓	✓	✓	✓	Increased under both stressors
MO_2_	✓	—	✓	—	Reduced by highest sediment (50 NTU) and warming
Total chlorophyll	—	—	—	—	Stable
Chlorophyll *a*	✓	—	—	—	Reduced only at 50 NTU
Symbiodiniaceae density	✓	—	—	—	Reduced only at 15 NTU
Soluble protein	✓	—	—	—	Reduced only at 15 NTU
Gross net calcification	—	—	—	—	Stable

During the suspended sediment × temperature experiment, elevated temperature significantly reduced ΔFv/Fm, while ΔYNPQ responded to suspended sediment, temperature, and their interaction. In contrast, gross net calcification and all biological endpoints remained stable, and respiration responded only to elevated temperature. Across both experiments, significant genotype effects persisted after accounting for experimental treatments, highlighting the importance of intraspecific variation as a contributor to coral physiological performance (Table 2).

## Discussion

This study demonstrates that acute exposure to elevated temperature produced broader physiological responses in *Orbicella faveolata* than suspended sediments, while photophysiological metrics consistently emerged as the earliest indicators of stress. Across both experiments, photophysiological metrics were the most responsive indicators of environmental stress, whereas metabolic responses were more limited and tissue, symbiont, and calcification endpoints remained largely unchanged over the 72-h exposure period. Elevated temperature produced broader physiological effects than suspended sediment, and no suspended sediment × temperature interaction was detected under the conditions tested. Collectively, these findings indicate that photophysiological measurements provide sensitive indicators of acute sublethal stress, while biomass-related and skeletal responses appear less responsive during short-term exposure. Because physiological endpoints were not measured continuously through time, this hierarchy should be interpreted as one of relative short-term sensitivity rather than a fully resolved temporal sequence of physiological impairment.

### Thermal stress effects on coral photophysiology

Elevated temperature produced the most consistent physiological responses observed in this study, primarily affecting coral photophysiology. Reductions in maximum photochemical efficiency (Fv/Fm), together with increased regulated non-photochemical energy dissipation (YNPQ), indicate altered energy allocation by the algal symbionts under thermal stress. Declines in Fv/Fm are widely recognized as an early indicator of impaired photosystem II function, and recent work has further emphasized its sensitivity for quantifying the onset and progression of acute thermal stress in coral holobionts (Gomez-Campo & Baums, 2025; Helgoe et al., 2024; Warner et al., 1999). Increased Y(NPQ) further indicates activation of photoprotective energy dissipation, suggesting that the photosynthetic apparatus responded rapidly to elevated temperature before detectable changes occurred in symbiont density, chlorophyll concentration, tissue condition, or calcification.

Elevated temperature produced the most consistent physiological responses observed in this study, primarily affecting coral photophysiology. Maximum photochemical efficiency (Fv/Fm) declined, while regulated non-photochemical energy dissipation Y(NPQ) increased, indicating altered energy allocation by the algal symbionts under thermal stress. Declines in Fv/Fm are widely recognized as an early indicator of impaired photosystem II function, and recent work has further emphasized its sensitivity for characterizing the onset and progression of acute thermal stress in coral holobionts (Gomez-Campo & Baums, 2025; Helgoe et al., 2024; Warner et al., 1999). Increased Y(NPQ) indicates activation of photoprotective energy dissipation, suggesting that the photosynthetic apparatus responded rapidly to warming before detectable changes occurred in symbiont density, chlorophyll concentration, tissue condition, or calcification.

This pattern is consistent with recent evidence that disruption of coral–Symbiodiniaceae function can begin before visible bleaching or substantial changes in conventional indicators of coral condition. Marangon et al. (2025) found that relatively low accumulated heat stress altered nutrient cycling and increased metabolic turnover in *Porites lutea*, shifting the holobiont toward net heterotrophy before pronounced declines in overall condition. Symbiodiniaceae also exhibited a stronger early molecular response than the coral host, supporting the view that symbiont-associated processes can be particularly sensitive during the initial stages of thermal stress. The rapid photophysiological responses observed in *O. faveolata* may therefore represent an early component of broader holobiont physiological disruption rather than an isolated response of the photosynthetic apparatus.

The increase in Y(NPQ), together with the absence of detectable changes in symbiont density, chlorophyll concentration, protein content, or calcification, is also consistent with activation of short-term photoprotective mechanisms. Such adjustments can dissipate excess excitation energy and limit photodamage before bleaching or structural deterioration becomes evident (Bhagooli & Hidaka, 2003; Warner et al., 1999). These responses therefore appear to reflect acute physiological regulation rather than severe impairment during the 72h exposure period.

Thermal effects nevertheless extended beyond photophysiology. Respiration also declined under elevated temperature, indicating changes in whole-colony metabolic performance. Recent synthesis of coral thermal-performance relationships suggests that, once thermal optima are exceeded, photosynthetic oxygen production can decline more rapidly than respiratory demand, potentially creating an imbalance between energy acquisition and metabolic requirements within the holobiont (Parry et al., 2025). Reduced metabolic performance is also consistent with disruption of photosynthetic activity and altered exchange between the coral host and its algal symbionts (Lesser, 1997; Warner et al., 1999). However, the comparatively modest metabolic response observed here, together with the stability of tissue-based endpoints and calcification, suggests that the 72h exposure captured an early stage of physiological stress before broader structural effects became detectable. These findings therefore support a hierarchy of short-term sensitivity in which photophysiological regulation responds more readily than whole-colony metabolism, tissue condition, or skeletal growth.

Significant genotype effects were also detected for several photophysiological metrics, highlighting substantial intraspecific variation in the response of *O. faveolata* to thermal stress. Such variability may reflect differences in host genotype, Symbiodiniaceae communities, or other intrinsic physiological characteristics and underscores the importance of incorporating multiple genotypes into experimental studies to better represent the range of stress responses present within natural populations.

### Effects of suspended sediment on coral physiology

In contrast to elevated temperature, exposure to suspended sediment produced relatively limited physiological responses during the 72h exposure. Although turbidity altered several photophysiological metrics, including reductions in Fv/Fm and ETRmax and increases in YNPQ, these changes were accompanied by only modest effects on respiration and no detectable changes in calcification, symbiont density, chlorophyll concentration, protein content, or tissue condition. This pattern is consistent with recent acute exposure experiments in *Montipora capitata*, in which suspended terrigenous sediment and warming affected symbiont-associated physiological processes before detectable changes in host condition, suggesting that short exposures may initially affect the photosynthetic component of the holobiont before effects propagate to the coral host (Good et al., 2025).

The comparatively limited responses observed here should also be considered in relation to sediment source and composition. The present study used cryomilled, homogenized inorganic sediment to standardize particle characteristics and isolate the effects of suspended particles and associated turbidity while minimizing biological and chemical variability. In contrast, Serrano et al. (2024) demonstrated that the effects of suspended sediments on *O. faveolata* larvae depended strongly on sediment origin: sediments collected near the Port of Miami produced greater reductions in survival and subsequent settlement than sediments collected from the natal reef, and the two sediment sources supported distinct microbial communities. Similarly, acute sedimentation has recently been shown to restructure the surface-mucus microbiome of *Porites lobata*, with microbial changes persisting or becoming more pronounced after the sedimentation event (Nakoa et al., 2025). Together, these studies demonstrate that sediment exposure cannot be characterized by turbidity magnitude alone; sediment origin, particle characteristics, associated microbial communities, and other biological or chemical constituents may substantially modify coral responses.

These distinctions likely help explain why the responses observed in the present experiment were less severe than those reported under some natural or experimentally complex sediment exposures. Continuous water flow also limited sustained sediment accumulation and allowed active sediment rejection, while the 72h duration restricted the time over which energetic costs could accumulate. Thus, our findings should not be interpreted as indicating that suspended sediments are inherently benign. Rather, they isolate the acute physiological consequences of a standardized inorganic suspended-sediment exposure and show that photophysiological regulation responds before measurable changes in biomass-related or skeletal endpoints. Recent evidence of source-specific and microbiome-mediated sediment responses further emphasizes the need to consider sediment composition and exposure history when extrapolating controlled turbidity experiments to natural dredging or runoff events.

Collectively, these findings indicate that the earliest physiological consequences of short-term suspended sediment exposure occur primarily through changes in photosynthetic regulation. The coordinated responses of Fv/Fm, ETRmax, and YNPQ reinforce the value of photophysiological measurements as sensitive indicators of acute sediment stress, whereas biomass-related and skeletal endpoints appear comparatively less responsive over short exposure periods.

### Intraspecific variation influences coral stress responses

A consistent finding across this study was that genotype accounted for significant variation in multiple physiological response variables after accounting for suspended sediment and temperature treatments. Significant genotype effects were detected for photophysiological metrics, respiration, and calcification, indicating that intrinsic biological differences among colonies contributed substantially to physiological performance. Such intraspecific variability has been widely documented in reef-building corals and may reflect differences in host genetics, Symbiodiniaceae communities, microbial associates, colony morphology, and physiological acclimatization that collectively influence stress tolerance (Bay & Palumbi, 2014; Edmunds, 1994; Rowan, 2004).

These findings have important implications for both experimental design and coral restoration. Studies relying on only a few colonies may overestimate or underestimate treatment effects if selected genotypes are unusually tolerant or susceptible to stress. Incorporating multiple genotypes provides a more representative estimate of species-level responses while improving the ability to distinguish environmentally driven responses from naturally occurring biological variation. Likewise, restoration programs increasingly emphasize maintaining genetic diversity to enhance resilience under future environmental change (Baums, 2008; van Oppen et al., 2015). The variability observed among *O. faveolata* genotypes suggests that preserving this diversity may improve the adaptive capacity of restored populations exposed to sedimentation and marine heatwaves.

### Lack of suspended sediment × temperature interactions

One objective of this study was to determine whether suspended sediment modified the physiological response of *O. faveolata* to elevated temperature. Although turbidity has been proposed to either alleviate thermal stress through light attenuation or exacerbate stress by reducing photosynthetic energy acquisition and increasing sediment rejection costs (Anthony & Fabricius, 2000; Weber et al., 2012), suspended sediment did not measurably buffer or exacerbate the effects of elevated temperature under the conditions tested.

This result contrasts with the recent findings of Good et al. (2025), who reported a synergistic effect of elevated temperature and suspended terrigenous sediment on metabolic oxygen flux in *Montipora capitata* during an acute factorial exposure. In that study, combined warming and turbidity produced a greater metabolic effect than either stressor alone, while responses also varied among corals originating from watersheds with different environmental histories. The difference between these results and those observed for *O. faveolata* highlights the context dependence of multiple-stressor interactions. Species identity, environmental history, sediment properties, exposure intensity, and experimental conditions may all influence whether suspended sediment amplifies, mitigates, or has little effect on thermal stress.

Accordingly, the absence of an interaction in the present study should not be interpreted as evidence that warming and sediment exposure act independently under all reef conditions. Rather, it indicates that a 29 NTU treatment of standardized inorganic sediment did not measurably alter the thermal sensitivity of *O. faveolata* during this acute exposure. More complex sediment sources, prolonged or repeated exposure, and greater accumulation of energetic costs may produce different outcomes, particularly under field conditions where sediment deposition, organic enrichment, microbial activity, and fluctuating light occur simultaneously.

### Calcification, biological condition, and recovery

Together, the responses observed across endpoints support a hierarchy of short-term physiological sensitivity, with photophysiological metrics responding most consistently, metabolic responses being more limited, and biomass-related and skeletal endpoints remaining comparatively unchanged during the 72h exposure. This pattern is consistent with recent evidence that substantial changes in symbiont and holobiont function can occur before conventional indicators of colony condition reveal comparable impairment (Good et al., 2025; Marangon et al., 2025). The absence of a calcification response should therefore not be interpreted as an absence of physiological stress, but rather as evidence that skeletal growth remained comparatively stable over the acute exposure period.

Maximum photochemical efficiency (Fv/Fm) returned to pre-exposure levels within 4 weeks following removal of the experimental stressors, demonstrating recovery of this photophysiological endpoint under the conditions tested. However, recovery of Fv/Fm should not be interpreted as evidence of complete holobiont recovery. Manalili et al. (2025), for example, found that several physiological characteristics of Symbiodiniaceae continued to decline during the early recovery of thermally stressed *Acropora hyacinthus*, even as host gene expression shifted from stress responses toward repair and metabolic reprogramming. These asynchronous responses demonstrate that recovery trajectories can differ among components and levels of biological organization. Thus, the return of Fv/Fm to pre-exposure values in *O. faveolata* indicates restoration of maximum photochemical efficiency. At the same time, the recovery trajectories of other physiological, cellular, and molecular processes remain unresolved.

### Implications for reef management

The inclusion of a 29 NTU suspended-sediment treatment provides physiological context for the turbidity magnitude historically used in Florida dredging permit compliance monitoring. Under the controlled laboratory conditions of this study, short-term exposure (72 h) to suspended inorganic sediment at this target turbidity altered coral photophysiology but did not produce detectable declines in calcification, tissue condition, or biomass-related metrics. These findings suggest that acute exposure to a management-relevant suspended sediment treatment may elicit early physiological stress before changes become apparent in more conventional indicators of coral health. Consequently, monitoring programs that rely primarily on colony condition, partial mortality, or growth measurements may fail to detect early sublethal physiological responses during short-term sediment exposure.

It is important to note that Florida’s historical turbidity criterion is expressed as 29 NTU above natural background. In contrast, the present study evaluated a target absolute turbidity of approximately 29 NTU under controlled laboratory conditions. Furthermore, this experiment examined a single coral species using one sediment source during an acute exposure period. Consequently, these findings should not be interpreted as validating or refuting existing regulatory criteria. Rather, they provide mechanistic insight into which physiological processes are most sensitive to acute suspended-sediment exposure and establish a biological framework for interpreting turbidity observations collected during coastal monitoring. Future evaluations of sediment-management criteria should continue to consider exposure duration, sediment characteristics, species-specific sensitivity, and field validation.

Photophysiological measurements may be particularly useful within such a framework. Recent studies have reinforced the sensitivity and ecological relevance of Fv/Fm, demonstrating its utility for detecting early loss of PSII function during acute thermal stress (Gomez-Campo & Baums, 2025) and showing that experimentally measured variation in photosystem efficiency can predict subsequent bleaching responses during natural thermal stress events (Caruso et al., 2025). Although these studies focused primarily on thermal stress, they provide independent evidence that photophysiological measurements can detect biologically meaningful changes before more conspicuous indicators of coral stress emerge. This is consistent with the present study, in which photophysiological responses were detected while tissue condition, biomass-related metrics, and calcification remained comparatively unchanged.

Because PAM fluorometry is rapid and non-destructive, incorporating it alongside water-quality measurements and visual colony assessments could improve detection of emerging physiological stress during dredging, coastal construction, and other sediment-generating activities. However, photophysiological stability or recovery should not be equated with whole-holobiont health, particularly given recent evidence that distinct physiological and molecular components can exhibit asynchronous stress and recovery trajectories. PAM fluorometry should therefore complement, rather than replace, conventional monitoring approaches. Integrating sensitive physiological indicators with measurements of turbidity, sediment characteristics, exposure duration, and colony condition would provide a more mechanistic framework for interpreting coral responses and could support earlier adaptive-management decisions before visible bleaching, tissue loss, or mortality occurs.

### Study limitations and future directions

Several considerations should be taken into account when interpreting these findings. First, the 72h exposure period was designed to evaluate acute physiological responses. It may have been insufficient to detect longer-term consequences such as bleaching, depletion of energy reserves, or reductions in calcification. Second, laboratory experiments cannot fully replicate the complexity of natural reef environments, where corals experience dynamic interactions among suspended sediments, sediment deposition, fluctuating light, water motion, nutrient availability, and other environmental stressors. Third, the study examined a single Caribbean reef-building coral species using standardized inorganic sediment under controlled laboratory conditions, and responses are therefore likely to vary among coral species, sediment characteristics, and environmental settings.

Future research should investigate how repeated and prolonged sediment exposure influences the progression from early photophysiological responses to changes in metabolism, tissue condition, and calcification. Integrating controlled laboratory experiments with long-term field monitoring across multiple coral species will be essential for establishing biologically meaningful sediment-management thresholds. Likewise, incorporating rapid physiological tools, such as PAM fluorometry, into field monitoring programs offers a promising approach to detecting sublethal coral stress before visible signs of impairment develop.

## Conclusion

This study demonstrates that acute exposure to elevated temperature produced broader physiological responses in *Orbicella faveolata* than in those exposed to suspended inorganic sediment. At the same time, photophysiological metrics consistently emerged as the earliest indicators of stress. Declines in Fv/Fm and shifts in non-photochemical quenching preceded detectable changes in respiration, tissue condition, or calcification, revealing a hierarchy of physiological sensitivity in which photosynthetic regulation responds before broader metabolic and structural processes. The significant influence of genotype across multiple physiological endpoints further underscores the importance of intraspecific variation in shaping coral resilience to environmental change.

Although the inclusion of a management-relevant 29 NTU suspended-sediment treatment provides valuable physiological context for interpreting short-term turbidity exposure, these controlled laboratory results should not be taken as validation of existing regulatory criteria. Instead, they establish a mechanistic framework for understanding how corals respond to acute suspended sediment and thermal stress and identify sensitive physiological metrics to detect early sublethal impacts. Integrating rapid, non-destructive tools such as PAM fluorometry into environmental monitoring programs could improve the early detection of coral stress, strengthen the biological interpretation of water-quality observations, and support more informed, science-based management of coral reefs facing increasing local and global stressors.

## Supplementary Information

Supplementary Material 1.

## Data Availability

Research data was generated in this study and can be found readily available here: https://github.com/CROH-Lab/Physiological-Thresholds
