# Supplementary material for: Hierarchical physiological sensitivity of the coral, Orbicella faveolata, to suspended sediment and elevated temperature
Source: Environ Monit Assess. 2026 Oct 13;198(11):1185. doi: 10.1007/s10661-026-15969-9 (PMC13661762; doi:10.1007/s10661-026-15969-9)
Supplement: Supplementary file 1 [file 10661_2026_15969_MOESM1_ESM.docx]

**Supplemental Information**

**Table S1.** **Experimental allocation of coral fragments across suspended sediment and temperature treatments.** Coral fragments from eight *Orbicella faveolata* genotypes were distributed among experimental chambers across four suspended sediment gradient trials and four suspended sediment × temperature trials. The suspended sediment gradient experiment included target turbidity treatments of 0, 15, 29, and 50 NTU at 27°C. The suspended sediment × temperature experiment used a fully crossed design with target turbidity treatments of 0 and 29 NTU at 27 and 30°C. The table lists the experiment, chamber assignment, genotype, fragment number, coral identification code, and target treatment conditions for each fragment.

|  | | | | | | **Target Treatment** | |
| --- | --- | --- | --- | --- | --- | --- | --- |
|  | **Exp** | **Chamber** | **Genotype** | **Fragment #** | **Coral-ID** | **Turbidity (NTU)** | **Temperature (°C)** |
| **Suspended Sediment Gradient** | 1.2 | 1 | 84 | 2 | 84-2 | 50 | 27 |
|  |  | 2 | 84 | 4 | 84-4 | 29 | 27 |
|  |  | 3 | 84 | 6 | 84-6 | 15 | 27 |
|  |  | 4 | 84 | 7 | 84-7 | 0 | 27 |
|  |  | 5 | 90 | 2 | 90-2 | 29 | 27 |
|  |  | 6 | 90 | 5 | 90-5 | 50 | 27 |
|  |  | 7 | 90 | 8 | 90-8 | 15 | 27 |
|  |  | 8 | 90 | 10 | 90-10 | 0 | 27 |
|  | 1.3 | 1 | 97 | 1 | 97-1 | 50 | 27 |
|  |  | 2 | 97 | 3 | 97-3 | 29 | 27 |
|  |  | 3 | 97 | 10 | 97-10 | 15 | 27 |
|  |  | 4 | 97 | 7 | 97-7 | 0 | 27 |
|  |  | 5 | 101 | 8 | 101-8 | 29 | 27 |
|  |  | 6 | 101 | 10 | 101-10 | 50 | 27 |
|  |  | 7 | 101 | 11 | 101-11 | 15 | 27 |
|  |  | 8 | 101 | 14 | 101-14 | 0 | 27 |
|  | 1.4 | 1 | 50 | 1 | 50-1 | 50 | 27 |
|  |  | 2 | 50 | 2 | 50-2 | 29 | 27 |
|  |  | 3 | 50 | 3 | 50-3 | 15 | 27 |
|  |  | 4 | 50 | 5 | 50-5 | 0 | 27 |
|  |  | 5 | 60 | 2 | 60-2 | 29 | 27 |
|  |  | 6 | 60 | 4 | 60-4 | 50 | 27 |
|  |  | 7 | 60 | 5 | 60-5 | 15 | 27 |
|  |  | 8 | 60 | 6 | 60-6 | 0 | 27 |
|  | 1.9 | 1 | 67 | 12 | 67-12 | 50 | 27 |
|  |  | 2 | 67 | 16 | 67-16 | 29 | 27 |
|  |  | 3 | 67 | 22 | 67-22 | 15 | 27 |
|  |  | 4 | 67 | 23 | 67-23 | 0 | 27 |
|  |  | 5 | 80 | 21 | 80-21 | 29 | 27 |
|  |  | 6 | 80 | 14 | 80-14 | 50 | 27 |
|  |  | 7 | 80 | 15 | 80-15 | 15 | 27 |
|  |  | 8 | 80 | 19 | 80-19 | 0 | 27 |
| **Suspended Sediment x Temperature** | 2.6 | 1 | 84 | 1 | 84-1 | 29 | 30 |
|  |  | 2 | 84 | 9 | 84-9 | 0 | 30 |
|  |  | 3 | 90 | 3 | 90-3 | 29 | 30 |
|  |  | 4 | 90 | 6 | 90-6 | 0 | 30 |
|  |  | 5 | 84 | 8 | 84-8 | 29 | 27 |
|  |  | 6 | 84 | 5 | 84-5 | 0 | 27 |
|  |  | 7 | 90 | 4 | 90-4 | 29 | 27 |
|  |  | 8 | 90 | 7 | 90-7 | 0 | 27 |
|  | 2.7 | 1 | 97 | 4 | 97-4 | 29 | 30 |
|  |  | 2 | 97 | 6 | 97-6 | 0 | 30 |
|  |  | 3 | 101 | 6 | 101-6 | 29 | 30 |
|  |  | 4 | 101 | 13 | 101-13 | 0 | 30 |
|  |  | 5 | 97 | 8 | 97-8 | 29 | 27 |
|  |  | 6 | 97 | 9 | 97-9 | 0 | 27 |
|  |  | 7 | 101 | 12 | 101-12 | 29 | 27 |
|  |  | 8 | 101 | 15 | 101-15 | 0 | 27 |
|  | 2.8 | 1 | 50 | 7 | 50-7 | 29 | 30 |
|  |  | 2 | 50 | 9 | 50-9 | 0 | 30 |
|  |  | 3 | 60 | 7 | 60-7 | 29 | 30 |
|  |  | 4 | 60 | 9 | 60-9 | 0 | 30 |
|  |  | 5 | 50 | 8 | 50-8 | 29 | 27 |
|  |  | 6 | 50 | 10 | 50-10 | 0 | 27 |
|  |  | 7 | 60 | 8 | 60-8 | 29 | 27 |
|  |  | 8 | 60 | 1 | 60-1 | 0 | 27 |
|  | 2.10 | 1 | 67 | 14 | 67-14 | 29 | 30 |
|  |  | 2 | 67 | 18 | 67-18 | 0 | 30 |
|  |  | 3 | 80 | 22 | 80-22 | 29 | 30 |
|  |  | 4 | 80 | 16 | 80-16 | 0 | 30 |
|  |  | 5 | 67 | 15 | 67-15 | 29 | 27 |
|  |  | 6 | 67 | 11 | 67-11 | 0 | 27 |
|  |  | 7 | 80 | 13 | 80-13 | 29 | 27 |
|  |  | 8 | 80 | 18 | 80-18 | 0 | 27 |

**Table S2. Physical and compositional characteristics of Port Everglades sediment used in the experimental exposures.** Initial sediment composition and grain-size values are mean ± SD (*n* = 3). Post-milling particle-size classes were calculated by combining the reported laser-diffraction bins; values may differ slightly from 100% because of rounding.

| **Sediment characteristic** | **Size range or component** | **Value (%)** |
| --- | --- | --- |
| **Initial composition** | Moisture | 0.48 ± 0.09 |
|  | Organic matter | 3.13 ± 0.41 |
|  | Carbonate fraction | 31.07 ± 1.89 |
|  | Terrigenous fraction | 65.32 ± 1.48 |
| **Initial grain-size distribution** | Gravel (>2,000 µm) | 4.40 ± 2.74 |
|  | Coarse sand (500–2,000 µm) | 67.43 ± 3.51 |
|  | Medium sand (250–500 µm) | 25.25 ± 1.44 |
|  | Fine sand (63–250 µm) | 2.68 ± 1.35 |
|  | Silt and clay (<63 µm) | 0.25 ± 0.08 |
| **Post-milling particle-size distribution** | <63 µm | 20.23 |
|  | 63–125 µm | 24.92 |
|  | 250–500 µm | 51.54 |
|  | 1,000 µm | 3.32 |
|  | 2,000 µm | 0 |

**Table S3**. **Treatment-level environmental conditions during the suspended sediment and suspended sediment × temperature experiments.** Measured turbidity, temperature, salinity, and dissolved oxygen are summarized for each treatment within each experimental trial. The suspended sediment gradient experiment included control, 15, 29, and 50 NTU target treatments maintained at 27°C. The suspended sediment × temperature experiment crossed control and 29 NTU turbidity treatments with target temperatures of 27 and 30°C. Values are presented as mean ± SE.

|  | **Experiment** | **Treatment** | **Measured turbidity (NTU)** | **Measured temperature (°C)** | **Salinity (ppt)** | **Dissolved oxygen (mg O₂ L⁻¹)** |
| --- | --- | --- | --- | --- | --- | --- |
| **Suspended Sediment Gradient** | 1.2 | Control (no added sediment) | 1.02 ± 0.04 | 26.84 ± 0.04 | 35.77 ± 0.06 | 6.58 ± 0.03 |
|  |  | 15 NTU | 18.75 ± 1.45 | 26.74 ± 0.03 | 35.77 ± 0.01 | 6.60 ± 0.03 |
|  |  | 29 NTU | 30.07 ± 1.05 | 26.71 ± 0.04 | 35.69 ± 0.04 | 6.57 ± 0.02 |
|  |  | 50 NTU | 52.47 ± 2.23 | 26.69 ± 0.04 | 35.63 ± 0.07 | 6.55 ± 0.06 |
|  | 1.3 | Control (no added sediment) | 0.93 ± 0.06 | 26.80 ± 0.07 | 35.88 ± 0.13 | 6.55 ± 0.02 |
|  |  | 15 NTU | 14.27 ± 0.71 | 26.74 ± 0.02 | 35.91 ± 0.02 | 6.55 ± 0.00 |
|  |  | 29 NTU | 27.06 ± 1.37 | 26.50 ± 0.05 | 35.91 ± 0.08 | 6.55 ± 0.04 |
|  |  | 50 NTU | 56.12 ± 2.14 | 26.55 ± 0.02 | 35.93 ± 0.01 | 6.56 ± 0.03 |
|  | 1.4 | Control (no added sediment) | 0.90 ± 0.06 | 26.68 ± 0.05 | 36.05 ± 0.12 | 6.54 ± 0.01 |
|  |  | 15 NTU | 14.76 ± 0.71 | 26.60 ± 0.04 | 36.09 ± 0.05 | 6.58 ± 0.02 |
|  |  | 29 NTU | 33.52 ± 1.67 | 26.37 ± 0.02 | 36.05 ± 0.03 | 6.58 ± 0.01 |
|  |  | 50 NTU | 49.47 ± 2.11 | 26.39 ± 0.05 | 36.10 ± 0.12 | 7.25 ± 0.64 |
|  | 1.9 | Control (no added sediment) | 1.46 ± 0.08 | 26.96 ± 0.05 | 35.67 ± 0.09 | 6.45 ± 0.04 |
|  |  | 15 NTU | 15.77 ± 0.84 | 26.93 ± 0.05 | 35.63 ± 0.18 | 6.36 ± 0.21 |
|  |  | 29 NTU | 24.52 ± 0.53 | 26.99 ± 0.05 | 35.69 ± 0.04 | 6.55 ± 0.03 |
|  |  | 50 NTU | 50.02 ± 1.84 | 26.94 ± 0.04 | 35.65 ± 0.02 | 6.64 ± 0.09 |
| **Suspended Sediment × Temperature** | 2.6 | Control, 27°C | 1.89 ± 0.06 | 27.03 ± 0.06 | 35.77 ± 0.09 | 6.53 ± 0.04 |
|  |  | Control, 30°C | 0.90 ± 0.06 | 29.99 ± 0.06 | 35.94 ± 0.07 | 6.09 ± 0.05 |
|  |  | 29 NTU, 27°C | 28.84 ± 0.56 | 27.11 ± 0.05 | 35.94 ± 0.07 | 6.49 ± 0.04 |
|  |  | 29 NTU, 30°C | 30.57 ± 0.56 | 30.00 ± 0.05 | 36.00 ± 0.14 | 6.06 ± 0.05 |
|  | 2.7 | Control, 27°C | 1.54 ± 0.03 | 27.20 ± 0.07 | 35.64 ± 0.07 | 6.41 ± 0.07 |
|  |  | Control, 30°C | 1.20 ± 0.03 | 30.01 ± 0.07 | 35.66 ± 0.17 | 6.10 ± 0.10 |
|  |  | 29 NTU, 27°C | 27.21 ± 1.10 | 27.21 ± 0.07 | 35.55 ± 0.15 | 6.44 ± 0.07 |
|  |  | 29 NTU, 30°C | 36.53 ± 1.10 | 29.89 ± 0.07 | 35.57 ± 0.18 | 6.17 ± 0.09 |
|  | 2.8 | Control, 27°C | 1.52 ± 0.04 | 27.25 ± 0.16 | 35.61 ± 0.16 | 6.47 ± 0.01 |
|  |  | Control, 30°C | 1.63 ± 0.04 | 30.12 ± 0.16 | 35.66 ± 0.09 | 6.02 ± 0.05 |
|  |  | 29 NTU, 27°C | 30.34 ± 0.79 | 27.38 ± 0.16 | 35.66 ± 0.09 | 6.48 ± 0.04 |
|  |  | 29 NTU, 30°C | 28.07 ± 0.79 | 30.22 ± 0.16 | 35.54 ± 0.43 | 6.06 ± 0.06 |
|  | 2.10 | Control, 27°C | 0.63 ± 0.02 | 27.38 ± 0.46 | 35.63 ± 0.07 | 6.40 ± 0.04 |
|  |  | Control, 30°C | 0.73 ± 0.02 | 29.82 ± 0.46 | 35.67 ± 0.04 | 6.00 ± 0.05 |
|  |  | 29 NTU, 27°C | 29.65 ± 0.59 | 27.40 ± 0.46 | 35.61 ± 0.15 | 6.30 ± 0.05 |
|  |  | 29 NTU, 30°C | 29.75 ± 0.59 | 29.81 ± 0.46 | 35.64 ± 0.08 | 6.04 ± 0.02 |

**Table S4. Experiment-level physiological, biochemical, skeletal, and photophysiological responses of Orbicella faveolata to suspended sediment and elevated temperature treatments.** Values are presented as mean ± SE for each treatment within each experimental trial. Response variables include Symbiodiniaceae density, skeletal density, chlorophyll a and c₂ concentrations, total chlorophyll, host protein, total chlorophyll per symbiont, net calcification (GNET), daytime and nighttime oxygen flux (MO₂), and pre- and post-stress pulse-amplitude-modulated fluorometry metrics, including α, maximum electron transport rate (ETRm), maximum quantum yield of photosystem II (Fv/Fm), and non-photochemical quenching yield [Y(NPQ)]. The suspended sediment gradient experiment included control, 15, 29, and 50 NTU treatments at 27°C, whereas the suspended sediment × temperature experiment crossed control and 29 NTU treatments with temperatures of 27 and 30°C.

|  | **Exp** | **Treatment** | **Symbiodiniaceae density (×10⁶ cells cm⁻²)** | **Skeletal density (g cm⁻³)** | **Chlorophyll *a* (µg cm⁻²)** | **Chlorophyll *c*₂ (µg cm⁻²)** | **Total chlorophyll (µg cm⁻²)** | **Host protein (mg cm⁻²)** | **Total chlorophyll per symbiont (pg cell⁻¹)** | **Average GNET (CaCO3 g⁻¹ h⁻¹)** | **Average Day MO2 (mg O₂ g⁻¹ h⁻¹)** | **Average Night MO2 (mg O₂ g⁻¹ h⁻¹)** | **Avg Pre-Stress PAM Alpha** | **Avg Pre-Stress PAM ETRm** | **Avg Pre-Stress PAM Fv/FM** | **Avg Pre-Stress PAM Y(NPQ)** | **Avg Post-Stress PAM Alpha** | **Avg Post-Stress PAM ETRm** | **Avg Post-Stress PAM Fv/FM** | **Avg Post-Stress PAM Y(NPQ)** |
| --- | --- | --- | --- | --- | --- | --- | --- | --- | --- | --- | --- | --- | --- | --- | --- | --- | --- | --- | --- | --- |
| **Suspended Sediment Gradient** | 1.2 | Control (no added sediment) | 23.99 ± 0.80 | 1.31 ± 0.05 | 20.39 ± 0.57 | 16.02 ± 2.00 | 36.42 ± 2.57 | 8.98 ± 0.85 | 1.52 ± 0.06 | -0.04 ± 0.16 | -0.07 ± 0.00 | 0.04 ± 0.00 | 0.23 ± 0.02 | 137.22 ± 18.95 | 0.65 ± 0.00 | 0.24 ± 0.01 | 0.25 ± 0.02 | 158.73 ± 4.34 | 0.67 ± 0.02 | 0.21 ± 0.04 |
|  |  | 15 NTU | 26.98 ± 0.01 | 1.39 ± 0.00 | 25.65 ± 0.32 | 20.62 ± 2.53 | 46.27 ± 2.22 | 8.92 ± 0.55 | 1.71 ± 0.08 | -0.20 ± 0.02 | -0.08 ± 0.00 | 0.04 ± 0.00 | 0.24 ± 0.01 | 132.08 ± 7.07 | 0.66 ± 0.01 | 0.30 ± 0.01 | 0.25 ± 0.01 | 151.03 ± 5.98 | 0.68 ± 0.01 | 0.25 ± 0.02 |
|  |  | 29 NTU | 22.85 ± 0.79 | 1.35 ± 0.10 | 20.82 ± 3.66 | 13.78 ± 1.89 | 34.60 ± 1.77 | 9.30 ± 1.51 | 1.52 ± 0.13 | -0.41 ± 0.01 | -0.09 ± 0.00 | 0.04 ± 0.00 | 0.25 ± 0.01 | 129.38 ± 13.72 | 0.66 ± 0.01 | 0.27 ± 0.05 | 0.26 ± 0.00 | 148.78 ± 18.10 | 0.68 ± 0.01 | 0.22 ± 0.02 |
|  |  | 50 NTU | 21.89 ± 2.42 | 1.43 ± 0.09 | 22.58 ± 2.12 | 16.04 ± 2.01 | 38.62 ± 4.13 | 9.51 ± 1.47 | 1.77 ± 0.01 | -0.57 ± 0.06 | -0.07 ± 0.00 | 0.04 ± 0.00 | 0.20 ± 0.03 | 129.77 ± 10.37 | 0.62 ± 0.02 | 0.30 ± 0.05 | 0.23 ± 0.01 | 148.69 ± 10.11 | 0.66 ± 0.01 | 0.26 ± 0.01 |
|  | 1.3 | Control (no added sediment) | 14.15 ± 6.06 | 1.55 ± 0.11 | 18.93 ± 2.77 | 21.27 ± 4.30 | 40.21 ± 7.07 | 7.12 ± 0.06 | 3.22 ± 0.88 | -0.46 ± 0.53 | -0.07 ± 0.00 | 0.04 ± 0.00 | 0.24 ± 0.01 | 101.97 ± 12.12 | 0.68 ± 0.02 | 0.20 ± 0.00 | 0.22 ± 0.03 | 105.24 ± 13.42 | 0.68 ± 0.01 | 0.20 ± 0.02 |
|  |  | 15 NTU | 21.43 ± 11.55 | 1.51 ± 0.11 | 24.51 ± 5.14 | 24.23 ± 8.64 | 48.74 ± 13.78 | 7.91 ± 1.20 | 2.72 ± 0.82 | 0.08 ± 0.10 | -0.08 ± 0.00 | 0.04 ± 0.00 | 0.22 ± 0.02 | 103.28 ± 4.12 | 0.70 ± 0.02 | 0.12 ± 0.05 | 0.23 ± 0.02 | 97.99 ± 10.17 | 0.68 ± 0.00 | 0.20 ± 0.01 |
|  |  | 29 NTU | 13.30 ± 1.47 | 1.44 ± 0.02 | 17.27 ± 4.92 | 12.70 ± 1.29 | 29.97 ± 6.21 | 6.53 ± 0.00 | 2.23 ± 0.22 | 0.08 ± 0.07 | -0.08 ± 0.00 | 0.04 ± 0.00 | 0.24 ± 0.00 | 107.60 ± 12.37 | 0.70 ± 0.03 | 0.12 ± 0.05 | 0.24 ± 0.01 | 104.29 ± 7.45 | 0.69 ± 0.02 | 0.17 ± 0.00 |
|  |  | 50 NTU | 19.73 ± 3.69 | 1.60 ± 0.14 | 28.24 ± 8.89 | 18.54 ± 3.43 | 46.78 ± 12.32 | 6.70 ± 0.09 | 2.58 ± 1.11 | -0.24 ± 0.10 | -0.06 ± 0.00 | 0.04 ± 0.00 | 0.24 ± 0.01 | 99.63 ± 9.46 | 0.71 ± 0.03 | 0.18 ± 0.01 | 0.23 ± 0.01 | 101.84 ± 11.12 | 0.67 ± 0.02 | 0.21 ± 0.02 |
|  | 1.4 | Control (no added sediment) | 25.64 ± 8.82 | 1.47 ± 0.07 | 35.63 ± 2.50 | 37.12 ± 7.88 | 72.75 ± 5.38 | 8.15 ± 0.06 | 3.30 ± 1.34 | -0.27 ± 0.17 | -0.07 ± 0.00 | 0.04 ± 0.00 | 0.24 ± 0.00 | 127.75 ± 7.27 | 0.68 ± 0.00 | 0.23 ± 0.06 | 0.23 ± 0.02 | 124.40 ± 3.91 | 0.67 ± 0.01 | 0.29 ± 0.04 |
|  |  | 15 NTU | 30.18 ± 5.24 | 1.37 ± 0.01 | 29.56 ± 0.63 | 22.10 ± 4.92 | 51.65 ± 5.55 | 8.38 ± 0.59 | 1.80 ± 0.50 | -0.39 ± 0.01 | -0.08 ± 0.00 | 0.04 ± 0.00 | 0.23 ± 0.01 | 114.28 ± 8.15 | 0.68 ± 0.00 | 0.26 ± 0.06 | 0.21 ± 0.00 | 109.26 ± 1.23 | 0.67 ± 0.00 | 0.31 ± 0.05 |
|  |  | 29 NTU | 22.50 ± 3.10 | 1.61 ± 0.27 | 27.25 ± 0.81 | 22.52 ± 0.30 | 49.77 ± 1.11 | 8.33 ± 1.36 | 2.25 ± 0.26 | -0.92 ± 0.49 | -0.08 ± 0.00 | 0.04 ± 0.00 | 0.22 ± 0.00 | 115.18 ± 22.39 | 0.70 ± 0.02 | 0.23 ± 0.07 | 0.24 ± 0.01 | 118.21 ± 15.46 | 0.68 ± 0.01 | 0.26 ± 0.06 |
|  |  | 50 NTU | 38.33 ± 0.01 | 1.44 ± 0.17 | 37.49 ± 6.82 | 24.42 ± 0.04 | 61.91 ± 6.87 | 10.33 ± 0.22 | 1.62 ± 0.18 | -0.40 ± 0.05 | -0.07 ± 0.00 | 0.04 ± 0.00 | 0.25 ± 0.01 | 139.83 ± 2.70 | 0.68 ± 0.00 | 0.26 ± 0.04 | 0.23 ± 0.02 | 128.15 ± 0.33 | 0.68 ± 0.01 | 0.29 ± 0.05 |
|  | 1.9 | Control (no added sediment) | 13.84 ± 0.52 | 1.17 ± 0.13 | 19.38 ± 2.14 | 21.87 ± 0.44 | 41.25 ± 1.71 | 5.43 ± 4.78 | 2.98 ± 0.01 | -0.39 ± 0.26 | -0.07 ± 0.00 | 0.04 ± 0.00 | 0.20 ± 0.04 | 111.87 ± 20.03 | 0.65 ± 0.03 | 0.30 ± 0.02 | 0.20 ± 0.01 | 107.89 ± 19.64 | 0.63 ± 0.01 | 0.26 ± 0.01 |
|  |  | 15 NTU | 13.53 ± 0.33 | 1.28 ± 0.09 | 17.18 ± 0.91 | 15.85 ± 0.38 | 33.03 ± 1.29 | 4.31 ± 3.17 | 2.45 ± 0.15 | -0.14 ± 0.02 | -0.08 ± 0.00 | 0.04 ± 0.00 | 0.23 ± 0.01 | 136.86 ± 45.80 | 0.67 ± 0.00 | 0.32 ± 0.02 | 0.20 ± 0.00 | 114.94 ± 30.11 | 0.69 ± 0.03 | 0.26 ± 0.02 |
|  |  | 29 NTU | 13.10 ± 1.89 | 1.52 ± 0.06 | 17.81 ± 3.13 | 16.05 ± 6.22 | 33.87 ± 9.35 | 7.96 ± 1.13 | 2.53 ± 0.35 | -0.21 ± 0.10 | -0.08 ± 0.00 | 0.04 ± 0.00 | 0.21 ± 0.02 | 104.77 ± 25.72 | 0.67 ± 0.00 | 0.30 ± 0.01 | 0.21 ± 0.03 | 104.30 ± 39.42 | 0.66 ± 0.03 | 0.27 ± 0.06 |
|  |  | 50 NTU | 14.49 ± 1.10 | 1.36 ± 0.22 | 21.97 ± 2.93 | 22.39 ± 6.30 | 44.35 ± 9.23 | 8.67 ± 0.34 | 3.03 ± 0.41 | -0.05 ± 0.05 | -0.07 ± 0.00 | 0.04 ± 0.00 | 0.20 ± 0.02 | 65.48 ± 53.18 | 0.67 ± 0.01 | 0.30 ± 0.04 | 0.21 ± 0.02 | 128.80 ± 15.34 | 0.65 ± 0.01 | 0.24 ± 0.02 |
| **Suspended Sediment × Temperature** | 2.6 | Control (no added sediment), 27°C | 15.28 ± 3.37 | 1.43 ± 0.11 | 20.40 ± 0.16 | 15.63 ± 1.24 | 36.03 ± 1.09 | 6.48 ± 0.57 | 2.50 ± 0.62 | -0.22 ± 0.00 | -0.00 ± 0.00 | -0.02 ± 0.00 | 0.23 ± 0.00 | 84.28 ± 18.00 | 0.67 ± 0.00 | 0.28 ± 0.03 | 0.20 ± 0.02 | 84.09 ± 3.53 | 0.66 ± 0.01 | 0.30 ± 0.03 |
|  |  | Control (no added sediment), 30°C | 11.53 ± 3.55 | 1.38 ± 0.04 | 20.09 ± 3.84 | 13.34 ± 0.60 | 33.43 ± 4.44 | 8.34 ± 2.31 | 3.07 ± 0.56 | -0.21 ± 0.00 | 0.01 ± 0.02 | 0.00 ± 0.02 | 0.26 ± 0.02 | 92.42 ± 7.64 | 0.70 ± 0.01 | 0.22 ± 0.08 | 0.22 ± 0.02 | 85.91 ± 11.31 | 0.68 ± 0.02 | 0.27 ± 0.05 |
|  |  | 29 NTU, 27°C | 14.09 ± 3.46 | 1.50 ± 0.32 | 16.93 ± 0.30 | 15.94 ± 1.67 | 32.87 ± 1.97 | 8.67 ± 0.98 | 2.52 ± 0.76 | -0.21 ± 0.00 | 0.01 ± 0.00 | -0.01 ± 0.00 | 0.22 ± 0.00 | 82.06 ± 14.86 | 0.67 ± 0.00 | 0.33 ± 0.07 | 0.24 ± 0.01 | 83.32 ± 12.06 | 0.67 ± 0.02 | 0.30 ± 0.03 |
|  |  | 29 NTU, 30°C | 16.50 ± 0.66 | 1.56 ± 0.08 | 15.13 ± 1.13 | 10.26 ± 0.79 | 25.39 ± 1.92 | 6.45 ± 0.18 | 1.54 ± 0.05 | -0.20 ± 0.00 | 0.02 ± 0.00 | 0.01 ± 0.00 | 0.25 ± 0.02 | 81.11 ± 15.06 | 0.69 ± 0.00 | 0.25 ± 0.03 | 0.22 ± 0.00 | 72.15 ± 7.54 | 0.68 ± 0.01 | 0.31 ± 0.04 |
|  | 2.7 | Control (no added sediment), 27°C | 29.66 ± 12.22 | 1.35 ± 0.10 | 34.76 ± 13.90 | 23.80 ± 8.03 | 58.56 ± 21.93 | 8.55 ± 1.16 | 2.01 ± 0.09 | -0.26 ± 0.00 | 0.02 ± 0.01 | -0.01 ± 0.01 | 0.26 ± 0.02 | 119.86 ± 5.73 | 0.62 ± 0.01 | 0.33 ± 0.08 | 0.30 ± 0.05 | 105.00 ± 27.75 | 0.65 ± 0.03 | 0.33 ± 0.17 |
|  |  | Control (no added sediment), 30°C | 23.59 ± 0.37 | 1.51 ± 0.15 | 25.45 ± 1.79 | 13.22 ± 1.07 | 38.67 ± 2.86 | 6.86 ± 0.77 | 1.64 ± 0.15 | -0.32 ± 0.01 | 0.01 ± 0.00 | -0.00 ± 0.00 | 0.26 ± 0.02 | 122.14 ± 4.22 | 0.67 ± 0.03 | 0.26 ± 0.06 | 0.34 ± 0.03 | 77.14 ± 9.06 | 0.67 ± 0.03 | 0.39 ± 0.12 |
|  |  | 29 NTU, 27°C | 15.88 ± 4.94 | 1.42 ± 0.07 | 30.08 ± 3.64 | 30.29 ± 0.54 | 60.38 ± 4.18 | 9.45 ± 2.96 | 4.30 ± 1.60 | -0.25 ± 0.01 | 0.03 ± 0.01 | 0.01 ± 0.01 | 0.29 ± 0.07 | 101.75 ± 19.64 | 0.63 ± 0.01 | 0.39 ± 0.13 | 0.25 ± 0.00 | 129.88 ± 6.68 | 0.66 ± 0.01 | 0.27 ± 0.02 |
|  |  | 29 NTU, 30°C | 29.53 ± 8.00 | 1.22 ± 0.13 | 38.47 ± 1.75 | 19.82 ± 0.87 | 58.29 ± 0.87 | 9.45 ± 0.23 | 2.12 ± 0.54 | -0.30 ± 0.00 | 0.02 ± 0.01 | -0.00 ± 0.01 | 0.23 ± 0.03 | 122.75 ± 16.16 | 0.65 ± 0.03 | 0.27 ± 0.07 | 0.27 ± 0.02 | 78.32 ± 25.27 | 0.65 ± 0.02 | 0.33 ± 0.18 |
|  | 2.8 | Control (no added sediment), 27°C | 19.94 ± 8.62 | 1.66 ± 0.03 | 24.73 ± 6.36 | 12.69 ± 1.36 | 37.42 ± 7.72 | 5.51 ± 0.61 | 2.10 ± 0.52 | -0.19 ± 0.00 | 0.01 ± 0.01 | -0.00 ± 0.01 | 0.24 ± 0.03 | 92.27 ± 1.15 | 0.70 ± 0.03 | 0.21 ± 0.06 | 0.22 ± 0.01 | 80.65 ± 4.36 | 0.68 ± 0.01 | 0.28 ± 0.04 |
|  |  | Control (no added sediment), 30°C | 13.30 ± 2.27 | 1.51 ± 0.08 | 18.50 ± 2.50 | 10.08 ± 0.60 | 28.58 ± 3.09 | 4.41 ± 0.10 | 2.17 ± 0.14 | -0.22 ± 0.00 | -0.01 ± 0.01 | -0.03 ± 0.02 | 0.26 ± 0.00 | 87.52 ± 1.53 | 0.70 ± 0.01 | 0.19 ± 0.01 | 0.21 ± 0.01 | 78.72 ± 10.69 | 0.67 ± 0.00 | 0.26 ± 0.02 |
|  |  | 29 NTU, 27°C | 18.09 ± 2.64 | 1.38 ± 0.01 | 21.89 ± 2.59 | 11.73 ± 1.42 | 33.62 ± 4.01 | 4.51 ± 1.16 | 1.87 ± 0.05 | -0.19 ± 0.00 | 0.00 ± 0.00 | -0.02 ± 0.00 | 0.25 ± 0.03 | 95.22 ± 5.05 | 0.68 ± 0.03 | 0.24 ± 0.06 | 0.23 ± 0.01 | 93.99 ± 9.70 | 0.66 ± 0.00 | 0.27 ± 0.02 |
|  |  | 29 NTU, 30°C | 12.86 ± 1.23 | 1.49 ± 0.03 | 19.59 ± 3.57 | 14.17 ± 0.65 | 33.76 ± 4.22 | 5.05 ± 0.24 | 2.62 ± 0.08 | -0.22 ± 0.00 | 0.00 ± 0.00 | -0.02 ± 0.01 | 0.26 ± 0.02 | 98.83 ± 11.61 | 0.69 ± 0.03 | 0.23 ± 0.04 | 0.22 ± 0.01 | 80.20 ± 8.14 | 0.67 ± 0.01 | 0.26 ± 0.01 |
|  | 2.10 | Control (no added sediment), 27°C | 31.12 ± 5.32 | 1.36 ± 0.06 | 38.21 ± 1.15 | 24.98 ± 0.83 | 63.19 ± 0.33 | 9.59 ± 0.61 | 2.09 ± 0.37 | -0.23 ± 0.00 | 0.03 ± 0.00 | 0.02 ± 0.00 | 0.23 ± 0.01 | 180.46 ± 30.01 | 0.66 ± 0.01 | 0.19 ± 0.04 | 0.20 ± 0.00 | 122.34 ± 6.28 | 0.65 ± 0.01 | 0.29 ± 0.08 |
|  |  | Control (no added sediment), 30°C | 33.16 ± 11.51 | 1.33 ± 0.01 | 41.68 ± 17.95 | 24.92 ± 12.49 | 66.59 ± 30.44 | 8.92 ± 0.45 | 1.92 ± 0.25 | -0.23 ± 0.00 | 0.00 ± 0.01 | -0.01 ± 0.00 | 0.24 ± 0.01 | 149.17 ± 7.46 | 0.66 ± 0.01 | 0.22 ± 0.06 | 0.20 ± 0.01 | 128.59 ± 8.44 | 0.66 ± 0.00 | 0.32 ± 0.03 |
|  |  | 29 NTU, 27°C | 39.83 ± 14.11 | 1.29 ± 0.02 | 32.81 ± 3.77 | 22.71 ± 0.40 | 55.52 ± 3.37 | 9.27 ± 0.98 | 1.56 ± 0.47 | -0.23 ± 0.01 | 0.03 ± 0.00 | 0.02 ± 0.00 | 0.25 ± 0.01 | 162.90 ± 24.33 | 0.66 ± 0.00 | 0.22 ± 0.01 | 0.22 ± 0.02 | 120.38 ± 0.95 | 0.66 ± 0.00 | 0.30 ± 0.03 |
|  |  | 29 NTU, 30°C | 24.05 ± 8.89 | 1.12 ± 0.02 | 24.33 ± 0.68 | 13.77 ± 0.77 | 38.10 ± 1.45 | 8.01 ± 0.98 | 1.81 ± 0.61 | -0.22 ± 0.00 | 0.01 ± 0.00 | -0.00 ± 0.01 | 0.24 ± 0.01 | 151.94 ± 15.53 | 0.65 ± 0.01 | 0.23 ± 0.02 | 0.21 ± 0.00 | 109.64 ± 1.73 | 0.65 ± 0.02 | 0.32 ± 0.06 |

**Supplementary Table S5.** Fixed-effect parameter estimates and Tukey-adjusted pairwise comparisons for linear models evaluating the effects of suspended sediment treatment and genotype on coral physiological responses during the suspended sediment gradient experiment. Treatment coefficients are relative to the control treatment (0 NTU), and genotype coefficients are relative to the reference genotype. Significant effects (p < 0.05) are shown in bold.

| **Response Variable** | **Effect / Contrast** | **Estimate** | **SE** | **t value** | **P value** |
| --- | --- | --- | --- | --- | --- |
| **Chlorophyll a** |  |  |  |  |  |
|  | Treatment (15 NTU) | -3.844 | 1.984 | -1.94 | 0.063 |
|  | Treatment (29 NTU) | -4.175 | 2.326 | -1.8 | 0.083 |
|  | Treatment (50 NTU) | -5.963 | 2.041 | -2.92 | **0.007** |
|  | Genotype 60 | 5.097 | 2.844 | 1.79 | 0.087 |
|  | Genotype 67 | -13.193 | 2.403 | -5.49 | **<0.001** |
|  | ... | ... | ... | ... | ... |
| **Chlorophyll c₂** |  |  |  |  |  |
|  | Treatment (15 NTU) | 4.362 | 3.292 | 1.33 | 0.196 |
|  | ... | ... | ... | ... | ... |
| **Symbiont density** |  |  |  |  |  |
|  | Treatment (15 NTU) | -5.985 | 2.475 | -2.42 | **0.023** |
|  | ... | ... | ... | ... | ... |
| **Fv/Fm** |  |  |  |  |  |
|  | Treatment (15 NTU) | 0.001 | 0.003 | 0.23 | 0.82 |
|  | Treatment (29 NTU) | -0.009 | 0.003 | -2.81 | **0.005** |
|  | Treatment (50 NTU) | -0.015 | 0.003 | -4.84 | **<0.001** |
|  | ... | ... | ... | ... | ... |
| **Tukey-adjusted pairwise comparisons** |  |  |  |  |  |
| **ETRmax** |  |  |  |  |  |
|  | 0 vs 15 NTU | 5.841 | 2.635 | 2.22 | 0.12 |
|  | 0 vs 29 NTU | 8.711 | 2.604 | 3.35 | **0.005** |
|  | 29 vs 50 NTU | -7.322 | 2.561 | -2.86 | **0.023** |
| **Fv/Fm** |  |  |  |  |  |
|  | 0 vs 29 NTU | 0.009 | 0.003 | 2.81 | **0.026** |
|  | 0 vs 50 NTU | 0.015 | 0.003 | 4.84 | **<0.001** |
|  | 15 vs 29 NTU | 0.009 | 0.003 | 3.04 | **0.013** |
|  | 15 vs 50 NTU | 0.016 | 0.003 | 5.07 | **<0.001** |
| **Y(NPQ)** |  |  |  |  |  |
|  | 0 vs 50 NTU | -0.03 | 0.006 | -4.9 | **<0.001** |
|  | 15 vs 50 NTU | -0.017 | 0.006 | -2.81 | **0.026** |
|  | 29 vs 50 NTU | -0.019 | 0.006 | -3.15 | **0.009** |

**Supplementary Table S6.** Fixed-effect parameter estimates and post hoc pairwise comparisons for linear mixed-effects models evaluating the effects of suspended sediment (0 and 29 NTU), temperature (27 and 30°C), genotype, and their interactions on coral physiological responses during the factorial experiment. Treatment coefficients are relative to the reference levels (0 NTU and 27°C). Significant effects (p < 0.05) are shown in bold.

| **Response Variable** | **Effect / Contrast** | **Estimate** | **SE** | **t value** | **P value** |
| --- | --- | --- | --- | --- | --- |
| **Alpha** |  |  |  |  |  |
|  | Turbidity (29 NTU) | 0.03 | 0.06 | 0.51 | 0.644 |
|  | Temperature (30°C) | 0.019 | 0.043 | 0.44 | 0.689 |
|  | Turbidity × Temperature | -0.001 | 0.002 | -0.53 | 0.635 |
|  | Genotype 60 | 0.219 | 0.151 | 1.45 | 0.155 |
|  | Genotype 67 | 0.532 | 0.12 | 4.42 | **<0.001** |
|  | ... | ... | ... | ... | ... |
| **ETRmax** |  |  |  |  |  |
|  | Turbidity (29 NTU) | 0.672 | 1.235 | 0.54 | 0.587 |
|  | Temperature (30°C) | -1.058 | 0.888 | -1.19 | 0.234 |
|  | Turbidity × Temperature | -0.028 | 0.043 | -0.66 | 0.513 |
|  | Genotype 60 | -30.912 | 3.766 | -8.21 | **<0.001** |
|  | ... | ... | ... | ... | ... |
| **Fv/Fm** |  |  |  |  |  |
|  | Turbidity (29 NTU) | 0 | 0.001 | 0.3 | 0.768 |
|  | Temperature (30°C) | -0.003 | 0.001 | -3.08 | **0.002** |
|  | Turbidity × Temperature | 0 | 0 | -0.49 | 0.627 |
|  | ... | ... | ... | ... | ... |
| **Y(NPQ)** |  |  |  |  |  |
|  | Turbidity (29 NTU) | 0.058 | 0.008 | 7.37 | **<0.001** |
|  | Temperature (30°C) | 0.049 | 0.008 | 6.25 | **<0.001** |
|  | Turbidity × Temperature | -0.049 | 0.011 | -4.44 | **<0.001** |
|  | ... | ... | ... | ... | ... |
| **Chlorophyll c₂** |  |  |  |  |  |
|  | Turbidity (29 NTU) | -0.63 | 3.848 | -0.16 | 0.872 |
|  | Temperature (30°C) | 2.388 | 3.848 | 0.62 | 0.542 |
|  | Turbidity × Temperature | 0.83 | 5.443 | 0.15 | 0.88 |
|  | ... | ... | ... | ... | ... |
| **Chlorophyll a** |  |  |  |  |  |
|  | Turbidity (29 NTU) | -0.176 | 5.517 | -0.03 | 0.975 |
|  | Temperature (30°C) | 5.351 | 5.517 | 0.97 | 0.343 |
|  | Turbidity × Temperature | 1.35 | 7.803 | 0.17 | 0.864 |
|  | ... | ... | ... | ... | ... |
| **Chlorophyll a per Symbiodiniaceae** |  |  |  |  |  |
|  | Turbidity (29 NTU) | 0.276 | 0.46 | 0.6 | 0.555 |
|  | Temperature (30°C) | -0.106 | 0.46 | -0.23 | 0.82 |
|  | Turbidity × Temperature | -0.484 | 0.651 | -0.74 | 0.465 |
|  | ... | ... | ... | ... | ... |
| **Protein Concentration** |  |  |  |  |  |
|  | Turbidity (29 NTU) | 1.083 | 1.116 | 0.97 | 0.343 |
|  | Temperature (30°C) | 0.981 | 1.116 | 0.88 | 0.389 |
|  | Turbidity × Temperature | -0.248 | 1.579 | -0.16 | 0.877 |
|  | ... | ... | ... | ... | ... |
| **Symbiodiniaceae Density** |  |  |  |  |  |
|  | Turbidity (29 NTU) | -1.97 | 5.297 | -0.37 | 0.714 |
|  | Temperature (30°C) | 1.114 | 5.297 | 0.21 | 0.835 |
|  | Turbidity × Temperature | 5.084 | 7.491 | 0.68 | 0.505 |
|  | ... | ... | ... | ... | ... |
| **Gross Calcification (Gnet)** |  |  |  |  |  |
|  | Turbidity (29 NTU) | 0.03 | 0.06 | 0.51 | 0.644 |
|  | Temperature (30°C) | 0.019 | 0.043 | 0.44 | 0.689 |
|  | Turbidity × Temperature | -0.001 | 0.002 | -0.53 | 0.635 |
|  | ... | ... | ... | ... | ... |
| **Respiration** |  |  |  |  |  |
|  | Turbidity (29 NTU) | -0.009 | 0.004 | -2.3 | 0.105 |
|  | Temperature (30°C) | -0.016 | 0.004 | -4.22 | **0.024** |
|  | Time of day (Night) | -0.02 | 0.001 | -28.42 | **<0.001** |
|  | Turbidity × Temperature | 0.012 | 0.005 | 2.25 | 0.11 |
|  | ... | ... | ... | ... | ... |
| **Post hoc pairwise comparisons** |  |  |  |  |  |
| **Fv/Fm** | 27°C vs. 30°C | -0.024 | 0.006 | -4.4 | **<0.001** |
| **Respiration** | Day vs. Night | 0.02 | 0.001 | 28.42 | **<0.001** |
|  | 27°C vs. 30°C | 0.01 | 0.003 | 3.72 | **0.034** |
|  | 0 NTU vs. 29 NTU | 0.003 | 0.003 | 1 | 0.391 |
| **Y(NPQ)** | 27°C vs. 30°C | -0.024 | 0.006 | -4.4 | **<0.001** |

**Figure S1. Turbidity profiles during the experimental exposures.** Measured turbidity recorded throughout the three-day exposure period for each experimental chamber. Dashed horizontal lines indicate the target turbidity treatments. **(A)** Suspended sediment gradient experiment, in which chambers were maintained at target turbidity levels of 0, 15, 29, and 50 NTU. **(B)** Suspended sediment × temperature experiment, in which chambers were maintained at target turbidity levels of 0 NTU (gold) or 29 NTU (teal) under ambient (27°C) and elevated (30°C) temperature treatments. Points represent individual turbidity measurements collected throughout each day of the experiment. Occasional spikes in turbidity reflect sediment resuspension events associated with maintaining target treatment levels.

**Figure S2. Temperature profiles during the experimental exposures.** Measured seawater temperatures recorded throughout the three-day exposure period for each experimental chamber. Dashed horizontal lines indicate the target treatment temperatures. **(A)** Suspended sediment gradient experiment, in which all chambers were maintained at an ambient target temperature of 27°C while receiving suspended sediment treatments of 0, 15, 29, or 50 NTU. (B) Suspended sediment × temperature experiment, in which chambers were maintained at target temperatures of 27°C (blue) or 30°C (purple) under control (0 NTU) or suspended sediment (29 NTU) treatments. Points represent individual temperature measurements collected throughout each day of the experiment.

**Figure S3:** Post-treatment recovery of maximum photochemical efficiency (ΔFv/Fm) of *Orbicella faveolata* following the recovery period of 4 weeks. The left panel shows corals exposed to the suspended sediment gradient (0, 15, 29, and 50 NTU) (A.), and the right panel shows corals exposed to the suspended sediment × temperature treatments: control (27°C, no added sediment), elevated suspended sediment (N; 29 NTU), elevated temperature (T; 30°C), and combined suspended sediment and elevated temperature (NT; 29 NTU + 30°C) (B.). Values are expressed as the change in Fv/Fm relative to pre-exposure measurements (ΔFv/Fm). Boxplots represent the median, interquartile range, and 1.5 × interquartile range, with individual colonies shown as colored points corresponding to genotype. The dashed red line indicates no change from pre-exposure values (ΔFv/Fm = 0). Treatments sharing the same uppercase letter are not significantly different (Tukey-adjusted, *p* > 0.05).

**Figure S4.** Changes in biological endpoints of Orbicella faveolata following 72 h exposure to suspended sediment treatments (0, 15, 29, and 50 NTU). Panels show changes relative to pre-exposure values (Δ) for (A) symbiont density, (B) chlorophyll a concentration, (C) chlorophyll a per Symbiodiniaceae cell, (D) total protein concentration, and (E) chlorophyll c₂ concentration. Boxplots represent the median, interquartile range (IQR), and 1.5 × IQR, with individual colonies shown as colored points corresponding to genotype. Black circles indicate treatment means, and the dashed red line denotes no change from pre-exposure values (Δ = 0). Treatments sharing the same uppercase letter are not significantly different (Tukey-adjusted, p > 0.05).

**Figure S5.** Changes in biological endpoints of Orbicella faveolata following 72 h exposure to the suspended sediment × temperature factorial treatments: Control (27°C, no added suspended sediment), suspended sediment (N; 29 NTU), elevated temperature (T; 30°C), and combined suspended sediment and elevated temperature (NT; 29 NTU + 30°C). Panels show changes relative to pre-exposure values (Δ) for (A) symbiont density, (B) chlorophyll a concentration, (C) chlorophyll a per Symbiodiniaceae cell, (D) total protein concentration, and (E) chlorophyll c₂ concentration. Boxplots represent the median, interquartile range (IQR), and 1.5 × IQR, with individual colonies shown as colored points corresponding to genotype. Black circles indicate treatment means, and the dashed red line denotes no change from pre-exposure values (Δ = 0). Treatments sharing the same uppercase letter are not significantly different (p > 0.05).

**Figure S6.** Net calcification rates (*G*<sub>net</sub>; µmol CaCO₃ g buoyant wt⁻¹ h⁻¹) of *Orbicella faveolata* during the 72 h experimental exposures. Suspended sediment experiment showing net calcification across the 0, 15, 29, and 50 NTU treatments on Days 1–3 (A.). Suspended sediment × temperature experiment showing net calcification for the Control (27°C, no added suspended sediment), suspended sediment (N; 29 NTU), elevated temperature (T; 30°C), and combined suspended sediment and elevated temperature (NT; 29 NTU + 30°C) treatments on Days 1–3 (B.). Boxplots represent the median, interquartile range, and 1.5 × interquartile range, with individual coral fragments shown as colored points corresponding to genotype. The dashed red line indicates zero net calcification; values below zero represent net dissolution. Treatments sharing the same uppercase letter are not significantly different (*p* > 0.05).
